# Strain resilient and self-healing nanocomposite conductors with ultralow sheet resistance

**DOI:** 10.1038/s41467-026-71851-9

**Published:** 2026-05-12

**Authors:** Ke-Xin Hou, Buyun Yu, Zhengyang Qian, Zong-Ju Chen, Peng-Fei Qiu, Kosei Sasaki, Lu Ju, Chao Zhang, Wei-Bing Lu, Takao Someya, Tomoyuki Yokota, Cheng-Hui Li

**Affiliations:** 1 State Key Laboratory of Coordination Chemistry, School of Chemistry and Chemical Engineering, Collaborative Innovation Center of Advanced Microstructures, Nanjing University, Nanjing, China; 2 Institute of Engineering Innovation, Graduate School of Engineering, The University of Tokyo, Tokyo, Japan; 3 Department of Electrical Engineering and Information Systems, The University of Tokyo, Tokyo, Japan; 4 State Laboratory of Millimeter Waves, School of Information Science and Engineering, Southeast University, Nanjing, China

**Keywords:** Electronic devices, Electrical and electronic engineering

## Abstract

Strain resilient nanocomposite conductors with self-healing ability are vital for skin-mounted devices and system-level bioelectronics, especially for wireless systems that require extremely low surface resistance and high stability of electromagnetic wave transmission. However, nanocomposite conductors inherently suffer from a trade-off between conductivity and flexible deformability or self-healing ability. Herein, a strain resilient and self-healing conductor (with ultralow sheet resistance of 10.8 mΩ/sq) is fabricated based on a well-designed polymer binder. It not only maintains outstanding stretchability (a break at elongation over 700%) and electrical stability during repeated stretching (1000 cycles) but also exhibits self-healing performance. On this basis, a flexible antenna with outstanding electromagnetic performance (realized gain of 5.25 dBi, nearly equivalent to that of copper-based devices), excellent self-healing ability is realized for wireless e-skin which can support instant communication exceeding 70 m. This approach overcomes the trade-off between high conductivity and excellent mechanical property in nanocomposite conductor and paves the way for completely self-healing flexible antenna towards wireless e-skins.

## Abstract (web summary)

Strain-resilient, self-healing conductors are needed for wireless e-skin but usually face a trade-off between conductivity and flexibility. Hou et al. design a polymer-based conductor with ultralow sheet resistance and use it to build a high-performance, self-healing flexible antenna.

## Introduction

Flexible electronics represent a new generation of devices that employ soft materials as substrate or circuits matrices. Their exceptional mechanical adaptability enables transformative applications in continuous health monitoring, advanced human-machine interfaces, and implantable medical devices^1–5^. As an essential component of flexible electronics, stretchable conductors provide pathways for integrating diverse electronic modules into flexible systems and ensuring efficient electromagnetic transmission^6–12^. An ideal stretchable conductor should have good conductivity to ensure efficient microwave transmission and minimal energy loss, while maintaining conductive stability under mechanical deformation such as stretching, bending, twisting to realize reliable operation in dynamic environments characteristic of wearable devices^13–15^. Moreover, flexible electronic devices will inevitably suffer mechanical damage due to repeated wear or accidental scratches during practical use, resulting in structure or even device failure. Self-healing capabilities can improve the durability and lifetime of flexible conductors by recovering functions after damage^16–20^. Therefore, it is vital to develop soft conductors with low resistance, strain resilient and self-healing abilities for flexible electronics.

However, designing strain resilient and self-healing conductors with low sheet resistance is fundamentally challenging. The most used strategy to synthesize intrinsically stretchable conductors is to dope conductive nanocomposites into soft polymer matrix^21–29^. But this approach has achieved only modest success, mainly constrained by the low stretchability and trade-off between conductivity and self-healing ability. For instance, we obtained an elastic conductor by uniformly dispersing single-walled carbon nanotubes (SWNTs) in a fluorinated polymer which could be stretched to 70%^30^. However, the disconnection of conductive fillers during the stretching process results in an abrupt increase in resistance. To enhance the conductive stability, a new approach utilizing thermal evaporation to deposit a thin Au film on the surface of a polyurethane-PDMS (polydimethylsiloxane) membrane substrate was proposed^31^. Although the obtained conductor could maintain stable conductivity, the maximum strain is approximately 30%. Meanwhile, Chen et al. proposed a unique interlocking strategy to improve the adhesion and stability of Au-PDMS electrode inspired by plant roots^32–34^, and they further significantly enhanced the strain of stretchable conductors up to 300% by depositing thin, wrinkled metal layers onto elastic substrates^6^. However, Au films do not exhibit self-healing property. Son et al. reported a self-healing nanocomposite with a conductivity to 1187 S/cm for ECG electrode, but the sheet resistance could not meet the requirement for microwave transmission^35^. To improve the conductivity of self-healing nanocomposite, liquid metal and ionic liquid as softer conductive fillers compared to metallic fillers have been utilized^36–40^. Nonetheless, the inherent fluidity poses significant challenges regarding leakage risks. Overall, an intrinsically soft conductive nanocomposite with the integration of high and stable conductivity, strain resiliency and especially self-healing ability has not been reported yet (Supplementary Table 1).

**Fig. 1:**
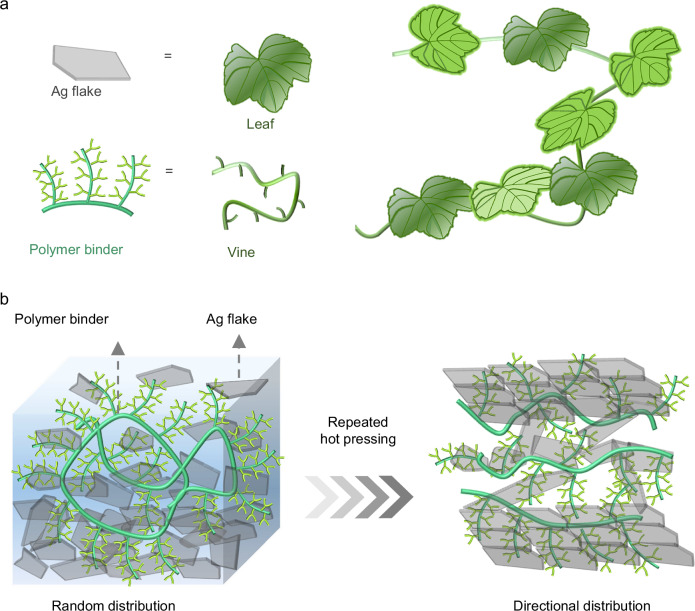
Fabrication of strain resilient nanocomposite conductor. **a** The role of Ag flakes and polymer binder in creeper-like nanocomposite conductors. **b** The fabrication process of strain-resilient conductors.

The conductivity of a conductive nanocomposite is dependent on the probability of filler contacts within the polymer matrix^12^. Therefore, low electrical resistance requires high loading conductive fillers, which will inevitably lead to increased rigidity of the composite material, propensity for cracking and difficulty to self-heal. Conductive fillers based on nanowires or nanoplates have the advantage of achieving enhanced percolation efficiency due to their high aspect ratios. Meanwhile, their structural anisotropy allows directional conductive pathways that improve both electrical conductivity and mechanical compliance in polymer composites. Moreover, it is reported that the platelike inorganic particles would migrate toward the surface of polymeric composites under thermal treatment due to the incompatibility of polymer materials and nanoparticles, leading to a surface-preferred distribution^41^. We therefore envisage that, by combining nanoplate conductive fillers and thermal-induced surface-preferred distribution process, a densely packed multi-layer stacking structure will be obtained. The conductive fillers behave like leaves of creeper, while the polymer binders function as the branches which can firmly interconnect the leaves to form a dense stacked structure (Fig. 1a). Benefiting from the interaction between the fillers and polymer binder, the nanocomposite would maintain stable conductivity under external mechanical environment. In this way, the inherent trade-off issue between conductivity and flexible deformability or self-healing capability, would possibly be addressed.

Herein, we designed and synthesized a brush polymer which can bind to metallic fillers with its electron-rich molecular chains. On this basis, an intrinsically soft, strain resilient, self-healing and highly stable nanocomposite, with multi-layer stacking structure, was fabricated. Owing to the spatiotemporal distribution of Ag flakes, it exhibited ultralow sheet resistance of 10.8 mΩ/sq, corresponding to conductivity up to 11574 S/cm. The intrinsically soft conductor not only maintains stable conductivity under strain exceeding 700% (Supplementary Fig. 1 and Supplementary Movie 1) but also shows ultra-stable electrical property over 1000 cycles at 50% strain. Moreover, benefiting from the viscoelastic property of the brush polymer binder, the conductive nanocomposite could realize instantaneous repair of electrical property and efficient self-healing of mechanical property. To demonstrate the outstanding electrical and physical performance of the stretchable conductor, we design and create a wearable antenna with excellent electromagnetic performance and self-healing ability for Bluetooth communication, and fabricate a wireless e-skin system for gesture recognition and human-machine interface which can realize stable wireless communication exceeding 70 m.

## Results and discussion

### Material design, synthesis and general characterization

The choice of suitable fillers and polymers is the key to the formation of multilayer interconnected conductive composites. The percolation threshold (the minimum volume fraction of conductive metallic filler required for a continuous electrical pathway in the composite) gradually decreases as the aspect ratio of the conductive filler increases, because fillers with larger aspect ratio are easier to form an interconnected conductive network^17^. On the other hand, the migration rate is related to the aspect ratio of the inorganic particles, with the smallest aspect ratio corresponds to the fastest migration rate^41^. Therefore, metal nanosheets with moderate aspect ratios are the best choice for the construction of conductive composites. Here, Ag flakes were chosen as the conductive fillers due to their ultralow contact resistance and superior anti-oxidation capability. As the other component of conductive nanocomposite, polymer binder is also very important. An ideal polymer binder should not only provide strong interactions with the filler to maintain electrical stability under high strain conditions but also have good flexibility and easy processability^42–44^. In this sense, brush polymer would be a good choice as the abundant branched chains can provide ample binding sites with metallic fillers, while its intrinsic softness can ensure low viscosity and promote the directional migration of Ag nanosheets to the material surface (Fig. 1b).

**Fig. 2:**
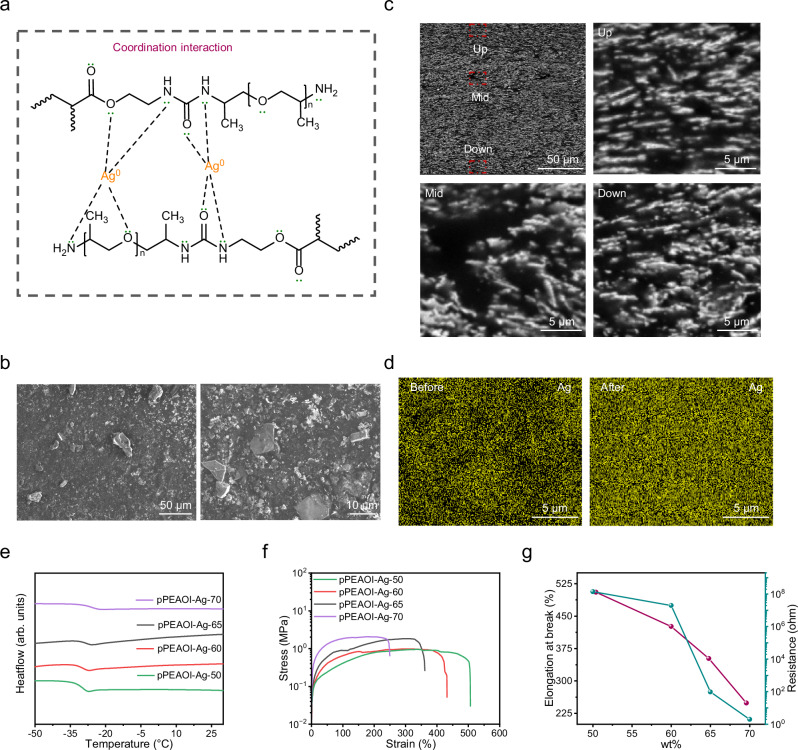
Design and characterization of conductive nanocomposites. **a** Coordination interactions between polymer binder pPEAOI and Ag flakes. **b** SEM images of nanocomposite conductor pPEAOI-Ag-70. **c** Cross-sectional SEM images and magnified images of different regions of nanocomposite conductor pPEAOI-Ag-70 after hot-pressing treatment. **d** The energy dispersive spectrometer (EDS) elements result of Ag in pPEAOI-Ag-70 before and after hot-pressing treatment. **e** DSC curves and **f** stress-strain curves of pPEAOI-Ag-X with different doped Ag flakes contents. **g** Change in elongation at break and bulk resistance (green axis) with different doped Ag flakes contents.

To ensure the strong interaction between polymer binder and Ag flakes, lone pair-rich polyetheramine (PEA) side chains are introduced into the brush polymer (Fig. 2a). The adhesive brush polymer is obtained by C = C polymerization of the prepolymer (Supplementary Fig. 2). Successful polymerization was evidenced by the proton nuclear magnetic resonance spectroscopy (^1^H NMR) (Supplementary Fig. 3) as well as Fourier transform infrared spectroscopy (FT-IR) (Supplementary Fig. 4). Based on successful synthesis the prepolymer PEAOI, a series of conductive composites pPEAOI-Ag-X (X represents the mass fraction of the doped Ag flakes) were obtained by mixing different mass fractions of Ag flakes with prepolymers. The obtained nanocomposites pPEAOI-Ag-X were then hot-pressed to promote the migration of Ag flakes to the surface (Fig. 2b). As shown in Fig. 2c, the scanning electron microscope (SEM) results of the cross-section show that there was a significant accumulation of Ag flakes in the area near the upper and lower surfaces compared with the middle region after hot pressing. The benefit of the post-treatment was further proved by energy dispersive spectrometer (EDS) elements analysis. As shown in Fig. 2d, the Ag element on the surface increased significantly after hot pressing. Notably, elemental O and elemental N exhibited the same behavior due to the interaction between Ag and lone pair electrons (Supplementary Fig. 5).

Subsequently, thermal gravimetric analysis (TGA) was conducted to characterize the thermal stability of the polymer binder and nanocomposite conductor. As shown in Supplementary Fig. 6, the resulting polymer pPEAOI and nanocomposite conductor pPEAOI-Ag-70 were thermally stable, with the decomposition temperature at 5% weight loss (*T*_*d5*_) of both samples are around 250 ± 10 °C. Additionally, differential scanning calorimetry (DSC) analysis was performed to evaluate the effect of the doped Ag flakes on the physicochemical property of polymer binder pPEAOI. As shown in Supplementary Fig. 7, the glass transition temperature (*T*_*g*_) of adhesive polymer pPEAOI is −32.67 °C. After doping Ag flakes, the glass transition temperature of conductive nanocomposite is slightly increased due to the strong interaction between polymer binder and Ag flakes. Specifically, the glass transition temperature of pPEAOI-Ag-50, -60, -65 and -70 is −30.98, −30.24, −29.16 and −27.24 °C, respectively (Fig. 2e). Additionally, FT-IR spectroscopy and X-ray photoelectron spectroscopy (XPS) test were conducted to confirm the coordination interaction between Ag flakes and polymer binder pPEAOI. As shown in Supplementary Fig. 8a, compared with the polymer pPEAOI, the absorption peaks of N-H, C = O, and C-O in the nanocomposite conductor pPEAOI-Ag-70 exhibit distinct red shifts which could be attributed to the donation of lone-pair electrons from nitrogen and oxygen atoms into the empty orbitals of Ag. As shown in Supplementary Fig. 8b, after the incorporation of pPEAOI, the Ag *3d*_3/2_ and Ag *3d*_5/2_ peaks shifted from 374.55 and 368.55 eV to 374.27 and 368.27 eV, respectively. Correspondingly, the peaks of N *1 s* and O *1 s* shifted to higher binding energy indicating the coordination interaction between Ag flakes and pPEAOI (Supplementary Fig. 8c, d). Benefiting from the flexibility of polymer chains, the conductive nanocomposites exhibited intrinsic stretchable behavior. As shown in Fig. 2f, the elongation at break of pPEAOI-Ag-50, -60, -65 and -70 is 505%, 426%, 352%, 249%, respectively, suggesting that the elongation at break of conductive composites decreased gradually with the increase of the doping amount of Ag flakes.

As the amount of doped Ag flakes increased from 50% to 70%, the bulk resistance of nanocomposite dropped sharply from 140 MΩ to 2 Ω (Fig. 2g). The dependence of sheet resistance of nanocomposite conductors on the different contents of doped Ag flakes was further measured by Four-probe Tester HPS2526 (Supplementary Fig. 9). An ultralow sheet resistance of 10.8 mΩ/sq, corresponding to a conductivity up to 11574 S/cm, was obtained for pPEAOI-Ag-70. Typical solid stretchable conductors have excellent conductivity properties, but their elongation at break is quite low due to the high fillers content. Surprisingly, pPEAOI-Ag-70 achieved a balance of high conductivity and flexible deformation ability (Supplementary Fig. 10) which is mainly due to the relatively high content of Ag flakes and the strong interaction between the polymer binder and the Ag flakes.

### Strain-insensitive electrical performance and self-healing properties

**Fig. 3:**
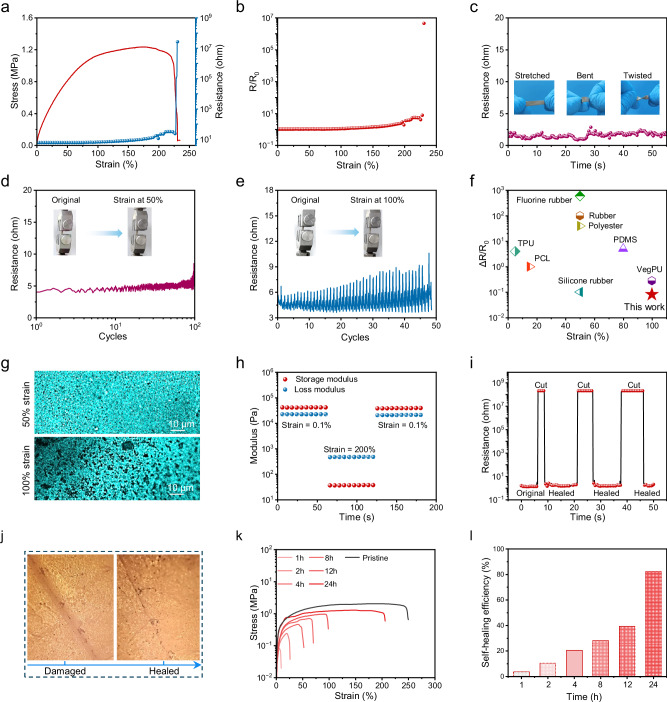
Strain-insensitive electrical performance, ultralow electrical hysteresis and self-healing property of pPEAOI-Ag-70. **a** Change in real-time resistance (blue axis) and stress of pPEAOI-Ag-70 under uniaxial stretching. **b** Relative resistance changes of pPEAOI-Ag-70 during uniaxial stretching. **c** Change in resistance of pPEAOI-Ag-70 under deformation such as stretched, twisted and bent. Change in resistance of pPEAOI- Ag-70 over **d** 100 stretch-relaxation cycles to 50% strain and **e** 48 stretch-relaxation cycles to 100% strain. **f** The comparison of cycling strain-electrical hysteresis among pPEAOI-Ag-70 and reported Ag-based conductors with various polymer binder including fluorine rubber^8^, rubber^49^, silicone rubber^50^, polyester^51^, PDMS^52^, PCL^53^, TPU^54^ and vegetable oil-based elastomers^27^. **g** The confocal images of pPEAOI- Ag-70 with the presence of 50% strain and 100% strain (bar: 10 μm). **h** Oscillation-time scan results of pPEAOI-Ag-70 at 25 °C, 1 Hz. **i** Instantaneous repair properties of electrical properties during the damaging-splicing process. **j** The self-healing process of pPEAOI-Ag-70 under optical microscope. **k** Stress-strain curves and **l** self-healing efficiency of pPEAOI-Ag-70 repaired at 80 °C for different periods.

The electrical stability of stretchable conductors is crucial for long-term stable operation of flexible electronic devices. However, conductive fillers sliding or microcracks usually generate when conductive composites are stretched, leading to a sharp increase in resistance. Notably, the conductive nanocomposite pPEAOI-Ag-70 exhibited strain insensitive conductivity. As shown in Fig. 3a, the bulk resistance of the conductive composite pPEAOI-Ag-70 remains very small until fracture. The extremely low ratio of real-time resistance to the original resistance (*R/R*_*0*_ = 1.10) upon stretching to 100% indicates that pPEAOI-Ag-70 has stable conductive property. (Fig. 3b). Conductivity-strain characteristics of the nanocomposite conductor pPEAOI-Ag-70 are shown in Supplementary Fig. 11. Moreover, as shown in Fig. 3c, pPEAOI-Ag-70 only exhibits slightly fluctuating resistance under stretched, twisted, bent and even punctured visually demonstrates the electrical stability of pPEAOI-Ag-70 under mechanical deformation conditions (Supplementary Movie 2).

The bulk resistance of pPEAOI-Ag-70 during multi-cycles stretch was measured to evaluate durability and electrical hysteresis. As shown in Fig. 3d, when the strain was fixed at 50%, the highest resistance of pPEAOI-Ag-70 was 8.48 Ω among nearly 100 cycles, and the change amplitude of resistance (*R-R*_*0*_) did not exceed 0.5 times of the original resistance value in 70 cycles (Supplementary Fig. 12a). In addition, when the fixed strain was 100%, the maximum bulk resistance of the conductive composite pPEAOI-Ag-70 only reached 10.8 Ω after nearly 50 cycles (Fig. 3e) which was only 2.5 times of the original resistance (Supplementary Fig. 12b). The electrical hysteresis performance (Δ*R*/*R*_0_, where Δ*R* = *R-R*_*0*_, *R* represents the resistance after cyclic deformation) of recently reported similar conductive materials using different polymers as binders and Ag flakes as fillers are summarized in Fig. 3f. It can be seen that pPEAOI-Ag-70 exhibits ultra-low electrical hysteresis performance under large strains. Overall, pPEAOI-Ag-70 not only has strain-insensitive conductivity under 130% stretch (maximum *R*/*R*_0_ = 1.20) but also can maintain high conductivity after multiple cyclic stretching under large strain conditions.

To further verify the ultra-stable conductivity of pPEAOI-Ag-70, fluorescence confocal imaging was utilized to characterize the surface structure of samples after stretched to 50% and 100% strain, respectively. As shown in Fig. 3g, only a few microcracks appeared, indicating that the polymer binder effectively suppresses crack formation during deformation. Rheological tests were also conducted to investigate the formation and recovery of these microcracks. As shown in Supplementary Fig. 13, pPEAOI-Ag-70 exhibited elastic behavior under small strain. When the strain reached 52%, both the storage and loss moduli dropped sharply, suggesting structural damage and the possible formation of microcracks. Notably, these microcracks could recover instantly. As illustrated in Fig. 3h, under 200% strain, the storage modulus was much lower than the loss modulus, confirming structural damage. However, when the strain recovered from 200 to 0.1%, the modulus immediately returned to its original value, indicating rapid crack recovery. This instant self-healing behavior is closely related to the structure and mechanical properties of the pPEAOI binder.

Besides stable electrical performance, pPEAOI-Ag-70 also exhibits self-healing behavior of electrical property and mechanical property. As shown in Fig. 3i, the bulk resistance could instantaneously recover after splicing at the fracture interface. By adding pPEAOI-Ag-70 to the conductive circuit, the instantaneous lighting of a small bulb can also be utilized as a reference for the repair of electrical property (Supplementary Fig. 14). Additionally, the scar on the surface of pPEAOI-Ag-70 disappeared after treatment at 80 °C for 24 h, indicating the self-healing performance of mechanical property (Fig. 3j). Stress-strain tests on samples repaired for different periods were performed to further quantify the self-healing efficiency of mechanical properties. As shown in Fig. 3k, the damaged splines could restore the stretchability after repair at 80 °C for 24 h, and the self-healing efficiency reached 82.49% (Fig. 3l). Overall, benefiting from the strong interaction between polymer binder pPEAOI and Ag flakes, the conductive nanocomposite pPEAOI-Ag-70 exhibited superior electrical performance and self-healing property.

### Mechanism and performance optimization

**Fig. 4:**
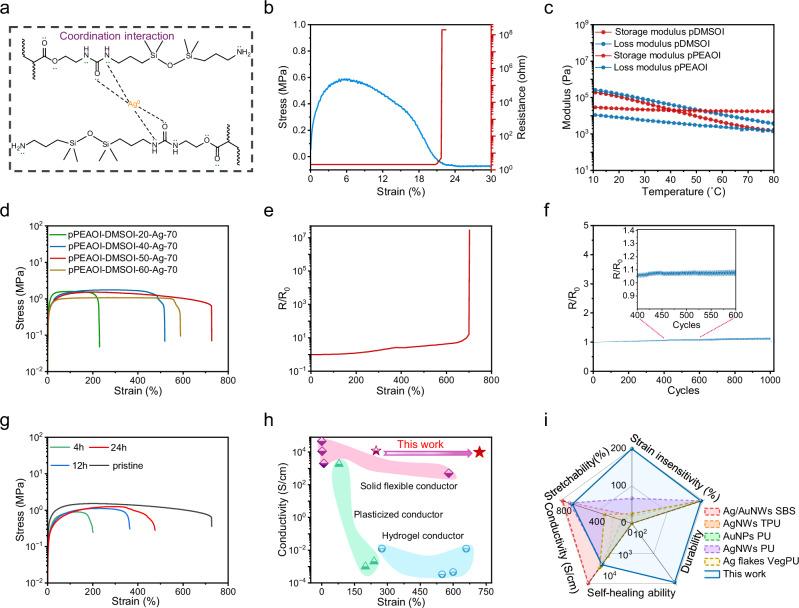
Mechanism and performance optimization of Ag flakes-based nanocomposite conductors. **a** Coordination interaction between pDMSOI and Ag flakes. **b** Change in real-time resistance (red axis) and stress of pDMSOI-Ag-70 under uniaxial stretching at 100 mm/min. **c** Temperature sweep scan results of pDMSOI and pPEAOI. **d** Stress-strain curves of conductive nanocomposites pPEAOI-DMSOI-y-Ag-70 with different DMSOI contents. **e** Relative resistance changes of pPEAOI-DMSOI-50-Ag-70 as a function of uniaxial strain. **f** Change in relative resistance of pPEAOI-DMSOI-50-Ag-70 over 1000 stretch-relaxation cycles to 50% strain, the small figure is a zoomed-in view of 400−600 cycles. **g** Stress-strain curves of damaged splines repaired at ambient condition for different periods. **h** Excellent conductivity and stretchability of the self-healing conductor compared with solid flexible conductor^55–58^ (red area), plasticized conductor^59–61^ (green area) and hydrogel conductor^62–65^ (blue area). **i** Comparison of comprehensive property of pPEAOI-DMSOI-50-Ag-70 to analogous reported metallic nanocomposites^17,21,24,27,66^.

To further demystify the strain-resilience and ultra-stable electrical performance of the nanocomposite conductors, control experiments were performed. As shown in Fig. 4a, PEAOI was replaced by DMSOI, leading to a similar brush polymer (pDMSOI) with reduced heteroatoms to prove the necessity of ether chains (Supplementary Fig. 15). ^1^H NMR spectrum (Supplementary Fig. S16) and FT-IR spectroscopy (Supplementary Fig. S17) confirmed the successful synthesis of DMSOI. Subsequently, Ag flakes were doped into pDMSOI by the same method as pPEAOI-Ag-70 to obtain the control sample pDMSOI-Ag-70. As shown in Fig. 4b, although pDMSOI-Ag-70 exhibited highly stable electrical property before fracture, the strain only reached about 20%. Rheological tests were conducted to explore the contribution of viscoelastic property to the stretchability of conductive nanocomposite. As shown in Fig. 4c, in contrast to pPEAOI which show elastic behavior, the loss modulus of pDMSOI was always larger than storage modulus from 10 to 80 °C, suggesting viscous behavior. Moreover, the stress-strain curves of pDMSOI and pPEAOI further confirmed the difference of viscoelastic property (Supplementary Fig. 18). Therefore, in addition to the weaker interaction between pDMSOI and Ag flakes, the poor stretchability of pDMSOI-Ag-70 may also be due to the viscous behavior of pDMSOI which could not provide sufficient support to the conductive fillers upon stretching.

Additionally, poly(ethylene glycol) methyl ether methacrylate (PEGMA) with a similar chemical structure which only has ether chains without urea bonds, was incorporated into the composite conductor as a polymer binder to demonstrate the necessity of urea bonds. The chemical structure of PEGMA is shown in Supplementary Fig. 19a. pPEGMA-Ag-70 was obtained after free radical polymerization by adding Ag flakes with a mass fraction of 70% to total mass. As shown in Supplementary Fig. 19b, the elongation of pPEGMA-Ag-70 is approximately 70%. During the stretching process, resistance of pPEGMA-Ag-70 increased from 39 to 1246 Ω (Supplementary Fig. 19c). Moreover, the relative resistance change remains stable only within a strain range of 13%. When the strain reaches 50%, the resistance reaches 179.86 Ω, correspondingly *R*/*R*_0_ = 4.61 (Supplementary Fig. 19d). Apparently, pPEGMA-Ag-70 exhibited obvious degradation of mechanical property accompanied by highly unstable electrical property during mechanical information due to the insufficient interaction between polymer binder and Ag flakes. Atomic Force Microscope (AFM) was utilized to further investigate the role of polymer binders in the migration and oriented distribution of Ag flakes. As shown in Supplementary Fig. 20, Ag flakes on the surface of pPEAOI-Ag-70 show obviously orderly packed distribution and lower roughness (*R*a = 40.6 nm), which have significant positive effects to the continuous and stable conductive pathways, thus resulting in ultralow sheet resistance and high conductivity. On the contrast, Ag flakes on the surface of pPEGMA-Ag-70 did not exhibit similar order distribution and pPEGMA-Ag-70 showed higher roughness (*R*a = 186.0 nm), leading to relatively low conductivity (55.80 S/cm). Therefore, we can propose a possible mechanism in which hot-pressing drives the immigration of Ag flakes from inside to the outside surface, at the same time, interaction between Ag flakes and polymer binder determines the order arrangement of the Ag flakes on the surface. Consequently, the percolation threshold is reduced. According to the classical percolation threshold theory, the predicted percolation threshold for our nanocomposite system is as low as 11.4% volume fraction (Supplementary Fig. 21), which is comparable to analogous studies with Ag flakes of similar size^8,35^.

The comparison between pPEAOI-Ag-70 and pDMSOI-Ag-70 provides valuable insights for optimizing the overall performance of conductive nanocomposites. While pPEAOI shows dominant elastic behavior from 10 to 80 °C and excellent resilience, its high elasticity limits chain mobility, reducing self-healing efficiency. In contrast, pDMSOI offers better self-healing performance due to the viscous behavior but lacks sufficient mechanical strength. To achieve a balance between stretchability and self-healing, a mixed polymer binder combining pPEAOI and pDMSOI was developed. In this system, pDMSOI functions as a plasticizer to enhance healing performance, while pPEAOI acts as a reinforcing phase to suppress filler slippage and crack formation under strain (Supplementary Fig. 22).

The resulting nanocomposites are denoted as pPEAOI-DMSOI‑y‑Ag‑70, where y is the mass ratio of DMSOI. Among the various formulations, pPEAOI-DMSOI-50-Ag-70 exhibits optimal performance (Fig. 4d). It achieves an elongation at break of 720%, and under 700% strain, it maintains remarkably low *R/R*_*0*_ values of only 15.8 which is the lowest among comparable systems even liquid metal-based conductors (Fig. 4e, Supplementary Table 1). The bulk resistance at 700% strain only reached 64.41Ω (Supplementary Fig. 23). Corresponding conductivity-strain characteristics of pPEAOI-DMSOI-50-Ag-70 are shown in Supplementary Fig. 24. As shown in Supplementary Fig. 25, due to the addition of viscous component pDMSOI, nanocomposite conductor pPEAOI-DMSOI-50-Ag-70 exhibited hysteresis behavior at small strain rates (Supplementary Fig. 26). The nanocomposite conductor pPEAOI-DMSOI-50-Ag-70 also demonstrates excellent fatigue resistance. As reported, natural human motions can induce strains up to 50% in skin-interfaced wearable systems during practical operation^45^. Therefore, to evaluate the stability of the nanocomposite conductor in practical applications, long-term cyclic stability testing was performed at a fixed tensile strain of 50%. As shown in Fig. 4f, after 1000 cycles at 50% strain, *R*/*R*_0_ stays below 1.15, and resistance remains under 3.85Ω (Supplementary Fig. 27), with only minor surface microcracks observed (Supplementary Fig. 28). Furthermore, maintaining a low resistance is essential for stretchable RF antennas, requiring high stability under strains up to 100%^42,46,47^. As shown in Supplementary Fig. 29, even at 100% strain, *R*/*R*_0_ stays below 1.13 over 70 cycles, with resistance under 4.28Ω, showing significantly improved performance over pPEAOI-Ag-70.

To directly prove the advantage of the directional architecture, we have replaced hot-pressing with ordinary cold-pressing during the post-treatment. A nanocomposite conductor with Ag flakes but without induced directional migration was fabricated and named as pPEAOI-DMSOI‑50‑Ag‑70-cold-pressing. As shown in Supplementary Fig. 30, pPEAOI-DMSOI‑50‑Ag‑70-cold-pressing exhibited a more pronounced increase in *R*/*R*_0_ with strain (maximum *R*/*R*_0_ = 1.92, compared to 1.15 for pPEAOI-DMSOI‑50‑Ag‑70), a significantly worse recovery of resistance when the strain is removed, and obvious electrical hysteresis after 1000 cycles deformation at strain of 50% (Δ*R*/*R*_0_ = 0.30). Furthermore, both tensile creep and shear creep experiments were conducted to evaluate the long-term stability of pPEAOI-DMSOI-50-Ag-70 as a component of wearable electronics under sustained tensile and shear stresses during service. As shown in Supplementary Fig. 31, it exhibited low creep strain value (13.20%) under small uniaxial stress (0.10 MPa) for 30 min and excellent recoverability after shear creep (from 25.35 to 3.21% at 3000 Pa), suggesting great potential in long-term stable wearable electronics. Importantly, the presence of viscous components lowers the self-healing temperature. After self-healing at ambient conditions for 24 h, healing efficiency can reach nearly 66% (Fig. 4g, Supplementary Fig. 32). In summary, through rational design of the polymer binder and tuning of viscoelastic properties, pPEAOI-DMSOI-50-Ag-70 achieves an excellent balance of stretchability and conductivity (Fig. 4h). It ranks among the most comprehensively high-performing intrinsically stretchable conductive composites reported (Fig. 4i).

### Intrinsically self-healable microwave devices for wireless e-skins

As the core component of wireless communication, microwave devices are widely used in e-skins, playing a crucial role in wireless communication and energy transfer. However, due to the propagating nature of electromagnetic waves, the performance of microwave devices is extremely sensitive to the conductivity and stability of conductors, and slight structural damage may interfere with wireless functions. Considering that wireless e-skins may encounter mechanical damage in practical applications, they should possess self-healing property. However, due to the stringent requirements on conductivity and stability, no microwave device that combines excellent electromagnetic performance and fully self-healable ability has been successfully reported. The physical properties of the nanocomposite conductor make it possible to realize intrinsically self-healable microwave devices with great electromagnetic performance, significantly advancing the development of wireless e-skins.

**Fig. 5:**
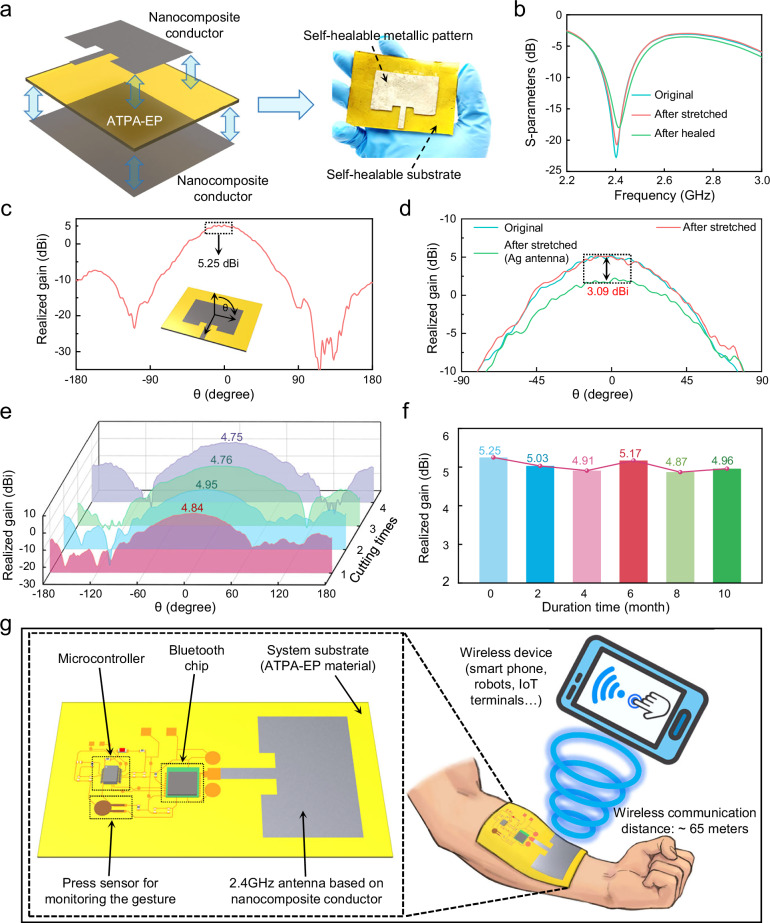
Stretchable and self-healable antenna based on nanocomposite conductor for wireless e-skins. **a** The integration process of the stretchable antenna. **b** The impedance matches characteristics of the antenna under the conditions of original state, stretching and releasing, and cutting and healing. **c** Measured radiation performance of the fabricated prototype. **d** The performance stability comparison between nanocomposite conductor-based antenna and Ag-based antenna. **e** The radiation performance of the nanocomposite conductor-based antenna after self-healable process. **f** The long-term stability of the nanocomposite conductor-based antenna. **g** The self-healable wireless electronic system based on nanocomposite conductor for human-machine interaction.

We designed and fabricated a stretchable antenna based on the nanocomposite conductor, supporting 2.4 GHz Bluetooth wireless communication. We constructed the antenna’s radiator and ground plane by the nanocomposite conductor and adopted the previously reported ATPA-EP material as the dielectric substrate^48^. Benefiting from the hydrogen bonds between the interface, they can integrate without any adhesive while avoiding excessive compatibility of interlayer structures (Supplementary Fig. 33). The fabricated microwave device is shown in Fig. 5a and the parameters of the metallic pattern are shown in Supplementary Fig. 34.

Firstly, we measured the impedance matching characteristics of the fabricated antenna. Reflection coefficient (*S*_*11*_) reveals the operational frequency of microwave devices and is closely related to the conductivity and stability of conductors. Experimental results demonstrate that the nanocomposite conductor-based antenna exhibits excellent impedance matching at 2.4 GHz, with the *S*_*11*_ as low as −23 dB (Fig. 5b). Additionally, thanks to the exceptional self-healing properties of nanocomposite conductor, the antenna demonstrates remarkable robustness against stretching and structural damage. As shown in Fig. 5b, after undergoing 20 cycles at 25% strain, the *S*_*11*_ of the antenna remains virtually unchanged. Furthermore, we cut the stretchable antenna along its central axis, carefully aligned the cut ends (Supplementary Fig. 35), and placed it for 10 min before testing. Owing to the instantaneous self-healing ability of electrical properties, the impedance matching of the antenna can restore to its initial state within a short period, with less than 1% difference in reflected electromagnetic energy even after complete slicing.

The radiation performance of an antenna is crucial for wireless e-skins, affecting communication range, signal quality, power use, and overall system design. Traditional antennas fail immediately if metal coils are damaged. Meanwhile, many self-healing conductive materials suffer from high resistance, limiting radiation efficiency. Our nanocomposite conductor, with ultralow sheet resistance, overcomes this issue. We tested the stretchable antenna’s radiation pattern in a microwave anechoic chamber (Fig. 5c). It achieved a high gain of 5.25 dBi, which is comparable to copper antennas, enabling stable long-range wireless communication. Thanks to the self-healing and stretch-insensitive properties, the antenna remains functional after repeated deformations. Compared to silver-paste antennas, the radiation efficiency is twice (3 dBi higher) under the same strain, due to the nanocomposite’s ability to reconstruct conductive paths (Fig. 5d). In extreme damage tests, we cut the antenna four times along its center and rejoined it. Even after four full cuts, it maintained a high gain of 4.75 dBi with minimal change in radiation pattern (Fig. 5e). Long-term tests also confirmed the stable performance overtime (Fig. 5f), showing strong potential for durable, high-performance wireless systems.

To further validate the real-world wireless communication ability of the antenna, we designed a wearable touch-interaction system, as illustrated in Fig. 5g and Supplementary Fig. 37. The wireless system shown in Supplementary Fig. 36 was fabricated on polyimide film and transferred onto the ATPA-EP substrate. The antenna was connected to the Bluetooth module and responsible for electromagnetic wave transmission between the module and a smartphone (Supplementary Movie 3). Experimental results demonstrate that the system achieves a maximum wireless communication range of 70–75 m, and it can continuously transmit the sensing data to the mobile phone (Supplementary Fig. 38). Additionally, we subjected the antenna of the wearable system to damage experiments and retested its wireless communication ability after self-healing. Experimental results indicate that after self-healing process, the maximum stable wireless communication range remains above 65 m, which is a quite impressive result (Supplementary Fig. 39). These findings highlight that our nanocomposite conductor not only ensures excellent electrical and electromagnetic performance but also significantly enhances the long-term reliability and stability of e-skin systems. Self-healable ability greatly helps to reduce the maintenance cost of wearable devices, minimize environmental pollution, and achieve sustainable development. Besides, Ag flakes in flexible antenna devices can be recycled after the processes of solvent dissolution, centrifugation, and drying (Supplementary Fig. 40). As shown in Supplementary Fig. 41, the nanocomposite conductor made from the recycled Ag flakes maintained stable conductivity under punctured, twisted, bent and stretched damage or deformation. Therefore, the nanocomposite conductor provides a technological foundation for the next generation of wireless e-skins and shows great potential in future wearable electronics, flexible communication systems, and biomedical applications.

In this study, we reported a strain-resilient conductor which not only can decouple mechanical properties from electrical properties but also can realize self-healing. The ultralow sheet resistance is primarily attributed to the hot pressing promoted directional migration of Ag nanosheets to the material surface, forming an interconnected multilayered structure. The interconnected conductive pathways, restrained by the polymer binder, avoid sliding and disconnecting under stretching conditions, thereby ensuring highly strain-insensitive electrical performance under nearly 200% strain and maintaining its electrical integrity even when subjected to strains exceeding 700%. An intrinsically self-healing antenna was fabricated utilizing the strain-resilient conductor which can maintain excellent performance under bent, stretched even after cut. Empowered by this novel antenna, the wireless e-skin system can support robust communication performance even under repeated mechanical deformations or damages, indicating enhanced transmission stability and prolonged service life. The fabrication strategy of stretchable conductors with superior electrical performance and self-healing ability promises intrinsically self-healing wireless wearable electronics.

## Methods

### Materials

Polyetheramine (D-400), 2,2’-Azobis(2-methylpropionitrile), 2-isocyanoethyl acrylate and 1,3-Bis(3-aminopropyl)-1,1,3,3-tetramethyldisiloxane were purchased from Aladdin Chemical Reagent Co., Ltd. Anhydrous dichloromethane were purchased from Energy Chemical Co., Ltd. Poly(ethylene glycol) methyl ether methacrylate (PEGMA, *n* = 9) was purchased from TCI (Tokyo Chemical Industry Co., Ltd., Shanghai). The Ag flakes were purchased from Jiangsu Xianfeng Nanomaterial Technology Co., Ltd., the diameter of the piece is 1–5 μm.

### Synthesis of PEAOI

4.000 g (0.0100 mol) of polyether amine (D-400) was weighed and placed in a 50 mL double-mouth bottle with 4 ml anhydrous methylene chloride. The oil pump was used to replace the gas in the reaction device with dry nitrogen, and the dynamic pumping is carried out three times in a row. At the same time, 1.424 g (1.010 eq, 0.0101 mol) of 2-isocyanethyl acrylate was weighed and injected into a 50 ml constant pressure dropping funnel with 15 mL anhydrous methylene chloride. The reaction device was placed in an ice water bath at 0 °C, and the solution of 2-isocyanethyl acrylate was slowly dropped to the bottle. After the dropwise addition was completed, the reaction device was moved to room temperature for 6 h, and then the methylene chloride was removed by rotary evaporator at 45 °C to PEAOI.

### Synthesis of DMSOI

2.485 g (0.0100 mol) of 1,3-bis(3-aminopropyl)tetramethyldisiloxane was weighed and placed in a 50 mL double-mouth bottle with 4 ml anhydrous methylene chloride. The oil pump was used to replace the gas in the reaction device with dry nitrogen, and the dynamic pumping is carried out three times in a row. At the same time, 1.424 g (1.010 eq, 0.0101 mol) of 2-isocyanethyl acrylate was weighed and injected into a 50 ml constant pressure dropping funnel with 15 mL anhydrous methylene chloride. The reaction device was placed in an ice water bath, and the solution of 2-isocyanethyl acrylate was slowly dropped to the bottle. After the dropwise addition was completed, the reaction device was moved to room temperature for 6 h, and then the methylene chloride was removed by rotary evaporator at 45 °C to obtain DMSOI.

### Synthesis of conductive nanocomposites

In this work, the synthesis of self-healing conductive materials in different proportions were involved, the following experimental procedure is described with a doping ratio of 70 wt% Ag flakes. 0.3000 g of PEAOI was weighed and placed in the mold of polytetrafluoroethylene, then 0.7000 g of Ag flakes and 0.0030 g (mass fraction of 1%) free radical polymerization initiator 2,2-azodiisobutyronitrile was added to the prepolymer. The solid-liquid mixture was stirred to ensure well mixed. The mixture was placed in a vacuum drying oven at 80 °C for solvent-free polymerization. After the reaction for 4 h, the elastomer material pPEAOI-Ag-70 was repeatedly heated and pressed at 80 °C to obtain nanocomposite conductor pPEAOI-Ag-70.

The synthesis of pPEAOI-DMSOI-y-Ag-70 with different DMSOI contents was consistent with the above synthesis steps. For example, pPEAOI-DMSOI-50-Ag-70 was synthesized as following steps. 0.1500 g of PEAOI and 0.1500 g DMSOI was weighed and placed in the mold of polytetrafluoroethylene, then 0.7000 g of Ag flakes and 0.0030 g (mass fraction of 1%) free radical polymerization initiator 2,2-azodiisobutyronitrile was added. The solid-liquid mixture was stirred to ensure well mixed. The mixture was placed in a vacuum drying oven at 80 °C for solvent-free polymerization. After reaction for 4 h, the material was repeatedly heated and pressed at 40 °C to obtain pPEAOI-DMSOI-50-Ag-70.

The synthesis of pPEGMA-Ag-70 with PEGMA (*n* = 9) was consistent with the above synthesis steps. 0.3000 g of PEGMA was weighed and placed in the mold of polytetrafluoroethylene, then 0.7000 g of Ag flakes and 0.0030 g (mass fraction of 1%) free radical polymerization initiator 2,2-azodiisobutyronitrile was added to the prepolymer. The solid-liquid mixture was stirred to ensure well mixed. After stirring, the mixture was placed in a vacuum drying oven at 80 °C for solvent-free polymerization. After reaction for 4 h, the material was repeatedly heated and pressed at 80 °C to obtain pPEGMA-Ag-70.

### Instruments and methods

The DSC test was performed using the PerKin Elmer Pyris 1 analyzer in a dry nitrogen atmosphere (50 mL/min) with a temperature range of −30–80 °C and a cooling and heating rate of 10 °C/min. ^1^H NMR spectra were obtained using a Bruker 400 MHz instrument. The ^1^H NMR spectrum is focused on the main structure, because telechelic (bifunctionally terminated) is only minor amount. The rheological properties were determined by a DHR-2 rheometer (TA Instruments). The temperature sweep test was performed at 1 Hz from 10 to 80 °C with a strain fixed at 0.1%. Mechanical properties were tested using Instron 3343. Scanning electron microscopy (SEM) images were acquired by a Hitachi S-4800 field emission microscope working at 5–10 kV. The self-healing performance evaluation was completed through a damaging-splicing-repair process. The damaged spline was left for different times under experimental conditions for self-healing, and then the same method as the original spline was used for stress-strain testing. Confocal fluorescence images were obtained using a confocal laser scanning microscopy (Carl Zeiss LSM 710) imaging system. The morphology of nanocomposite conductor was observed by atomic force microscopes (AFM, BrukerIcon). X-ray photoelectron spectroscopy was conducted by XPS (Thermo Scientific K-Alpha, USA).

### Electromagnetic simulation, measurement and circuit design

The full-wave simulation of the antenna was performed in CST 2024 Microwave Studio. The material property of the nanocomposite conductor was set as Ohmic Sheet according to the results of four-point probes. The impedance matching of the flexible antenna device was tested using the Rohde&Schwarz ZNB40 and Keysight N5227B vector network analyzer (VNA). The VNAs were calibrated before the *S*_*11*_ measurements. The flexible antenna was connected to the VNA via a coaxial cable and a SMA connector. The radiation pattern and realized gain of the flexible antenna device were measured in the microwave anechoic chamber in State Key Laboratory of Millimeter Waves. The digital circuit of the self-healable wireless system is designed in the Altium Designer (20.2.2), and it was fabricated on a 100 μm polyimide substrate by Shenzhen JLC group company. The components of the wireless system include 0805 footprint passive elements (resistors and capacitors), Ai-Thinker PB-01 Bluetooth module (this module has no antenna), STM32L031F6P6 microcontroller, RP-C5-ST press sensor, 1206 footprint LED indicator.

## Supplementary information

Supplementary Information

Description Of Additional Supplementary File

Supplementary Movie 1

Supplementary Movie 2

Supplementary Movie 3

Transparent Peer Review file

## Source data

Source data

## Data Availability

The main data supporting the findings of this study are available in the main text, the Supplementary Information, and the Source Data file. All other data from this work is available upon request from the corresponding author. Source data is provided in this paper. Source data are provided with this paper.
