# Supplementary material for: Strain resilient and self-healing nanocomposite conductors with ultralow sheet resistance
Source: Nat Commun. 2026 May 12;17:6344. doi: 10.1038/s41467-026-71851-9 (PMC13376812; doi:10.1038/s41467-026-71851-9)
Supplement: Supplementary file 1 — Supplementary Information [file 41467_2026_71851_MOESM1_ESM.pdf]

Supplementary Information

Strain Resilient and Self-healing Nanocomposite Conductors with Ultralow Sheet Resistance

Ke-Xin Hou[1,#], Buyun Yu[2,3,#], Zhengyang Qian[1], Zong-Ju Chen[1], Peng-Fei Qiu[1], Kosei Sasaki[2,3], Lu Ju[4], Chao Zhang[4], Wei-Bing Lu[4,*], Takao Someya[3,*], Tomoyuki Yokota[2,3,*], Cheng-Hui Li[1,*]

[1]State Key Laboratory of Coordination Chemistry, School of Chemistry and Chemical Engineering, Collaborative Innovation Center of Advanced Microstructures, Nanjing University, Nanjing 210023, China.

[2]Institute of Engineering Innovation, Graduate School of Engineering, The University of Tokyo, Tokyo 113-8656, Japan.

[3]Department of Electrical Engineering and Information Systems, The University of Tokyo, Tokyo 113-8656, Japan.

[4]State Laboratory of Millimeter Waves, School of Information Science and Engineering, Southeast University, Nanjing 210096, China.

*Correspondence to: chli@nju.edu.cn (C.-H. Li) or yokota@ntech.t.u-tokyo.ac.jp (T. Yokota) or someya@ee.t.u-tokyo.ac.jp (T. Someya) or wblu@seu.edu.cn (W.-B. Lu).

Supplementary Figures

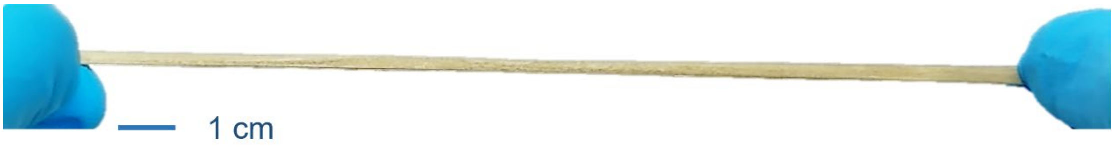

Supplementary Figure 1 Optical image of the nanocomposite conductor stretched exceeding 700% strain.

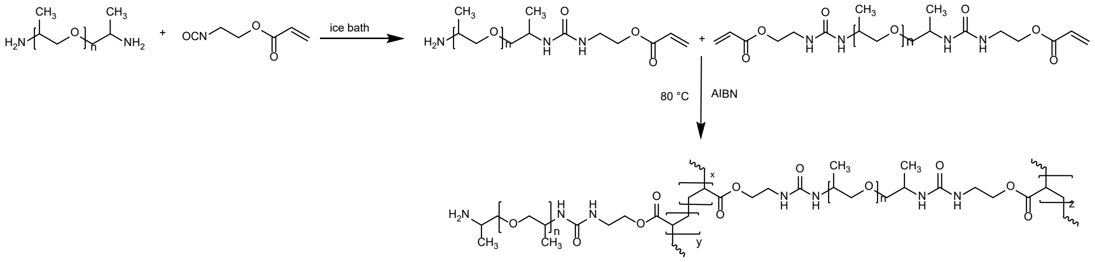

Supplementary Figure 2 The synthetic route polymer binder pPEAOI.

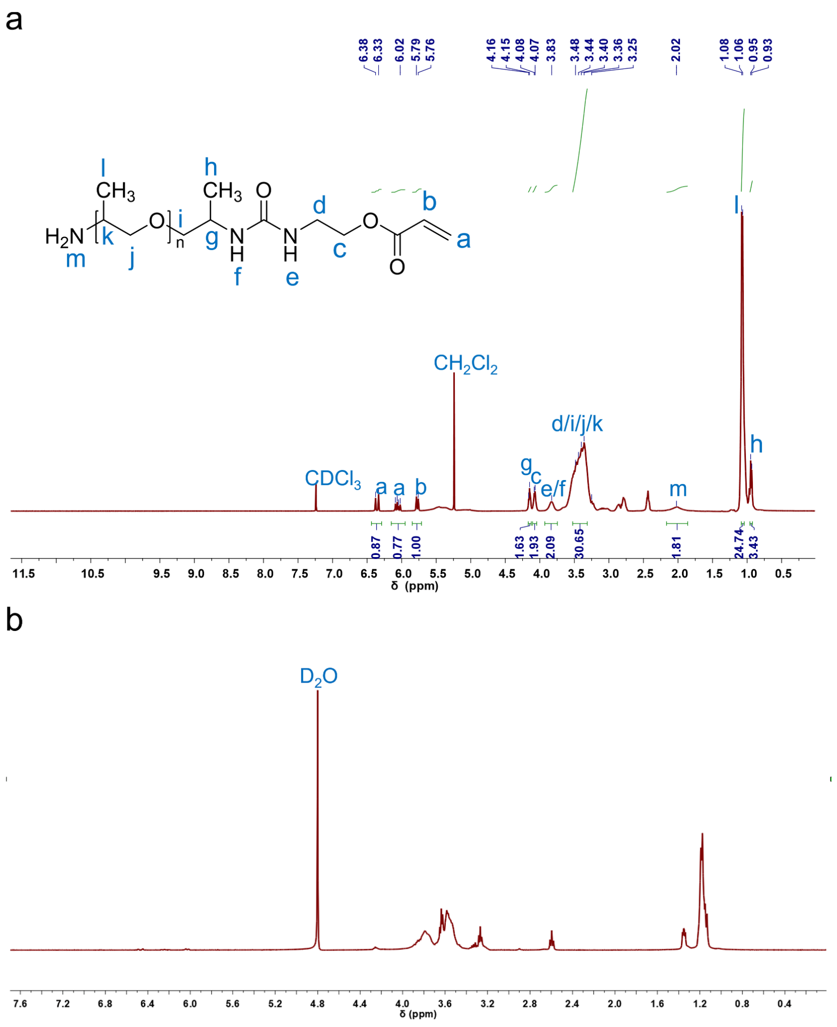

Supplementary Figure 3 ^{1}H NMR spectrum of (a) PEAOI: ^{1}H NMR (400 MHz, $CDCl_3$): δ (ppm) 6.36 (d, *J* = 17.3 Hz, 1H), 6.02 (m, 1H), 5.77 (d, *J* = 10.4 Hz, 1H), 4.16 (m, 1H), 4.07 (m, 2H), 3.83 (s, 2H), 3.40-3.25 (m, 22H), 2.02 (s, 2H), 1.07 (d, *J* = 5.7 Hz, 18H), 0.94 (d, *J* = 5.7 Hz, 3H). and (b) pPEAOI in D_2O.

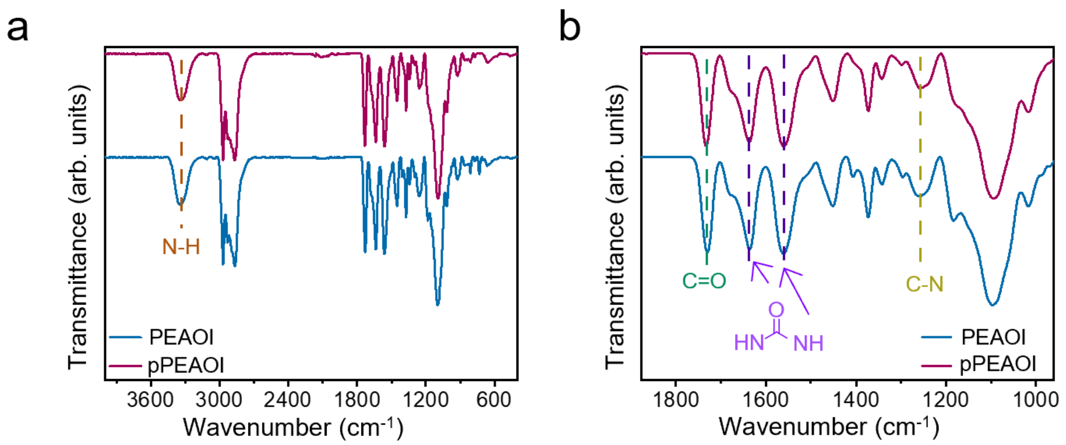

Supplementary Figure 4 Fourier transform infrared spectroscopy (FT-IR) of PEAOI and adhesive brush polymer pPEAOI from (a) 4000 to 400 cm^{-1} and (b) partial enlargement from 2000 to 900 cm^{-1}.

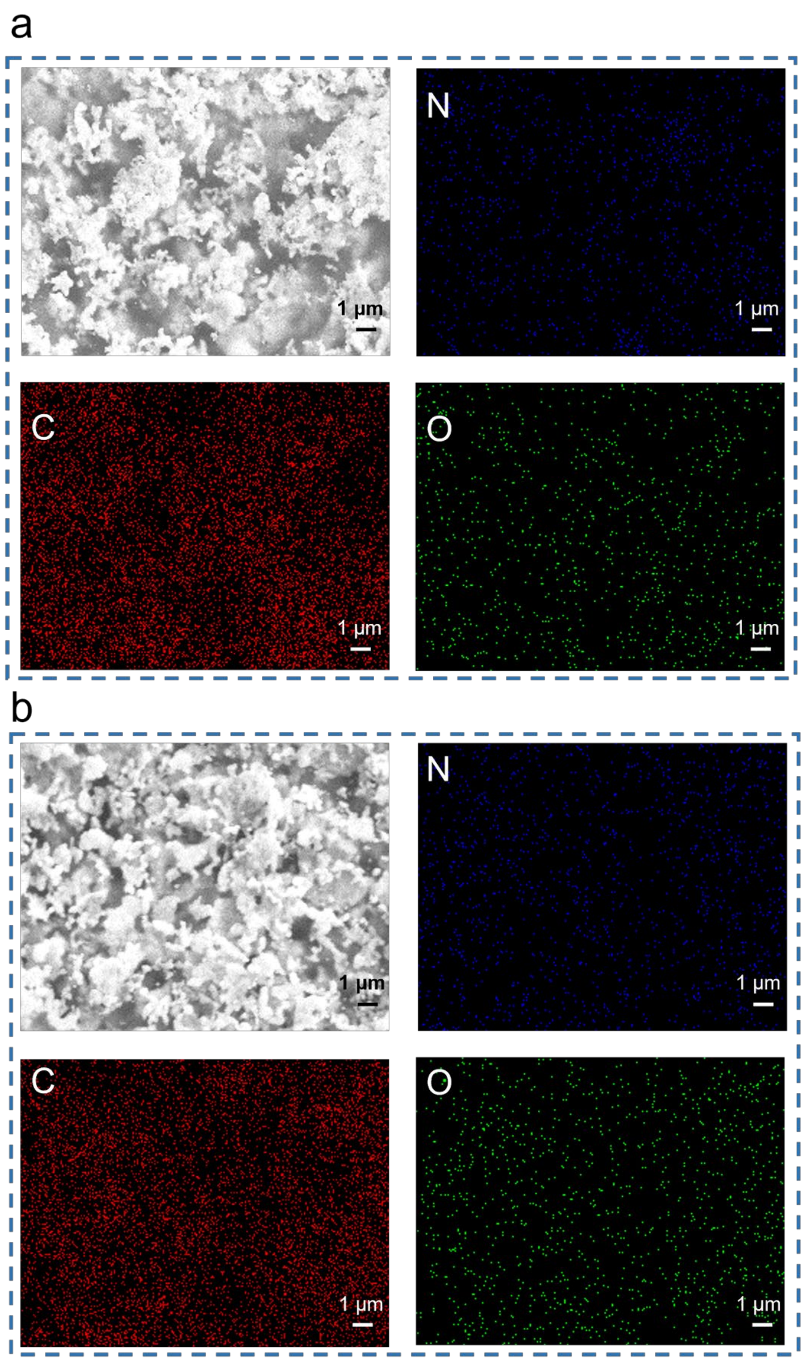

Supplementary Figure 5 The energy dispersive spectrometer (EDS) elements results of pPEAOI-Ag-70 (a) before and after (b) hot-pressing treatment.

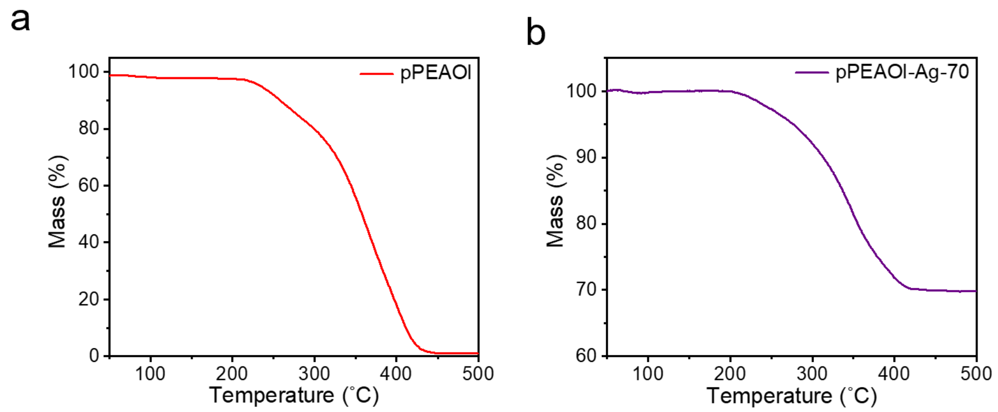

Supplementary Figure 6 TGA curves of (a) pPEAOI and (b) pPEAOI-Ag-70.

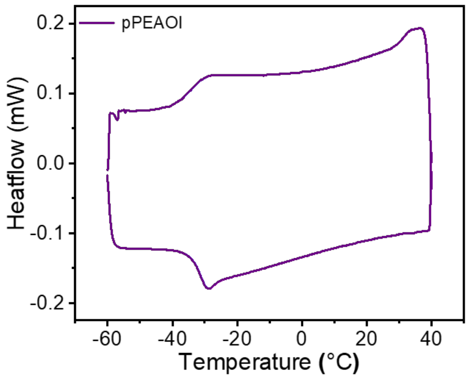

Supplementary Figure 7 DSC curve of polymer binder pPEAOI.

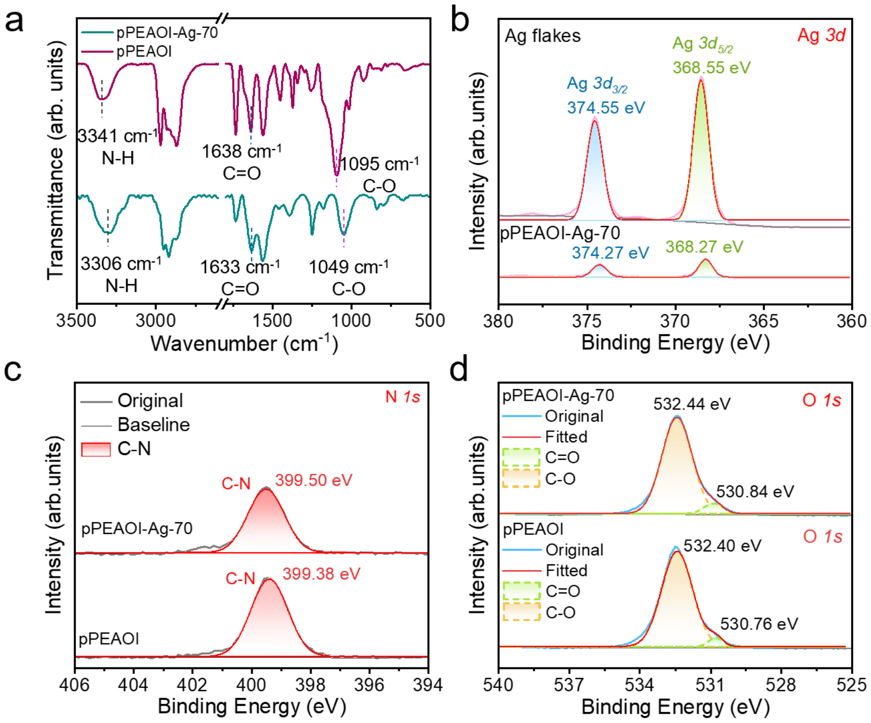

Supplementary Figure 8 Conformation of coordination interaction between polymer binder and Ag flakes. (a) FT-IR spectroscopy of pPEAOI and pPEAOI-Ag-70. (b) Ag *3d* core level XPS spectra of Ag flakes and pPEAOI-Ag-70. (c) N *1s* and (d) O *1s* core level XPS spectra of pPEAOI-Ag-70 and pPEAOI.

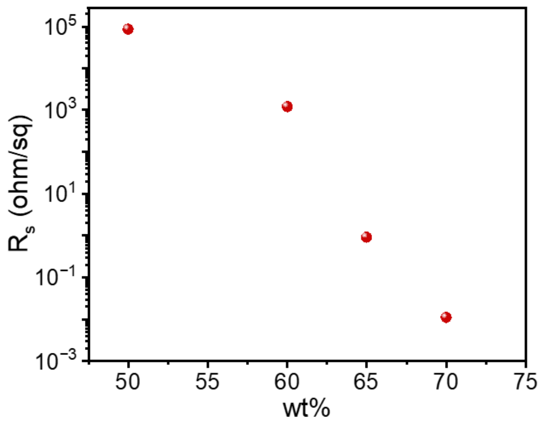

Supplementary Figure 9 Sheet resistance depends on weight percentage of Ag flakes.

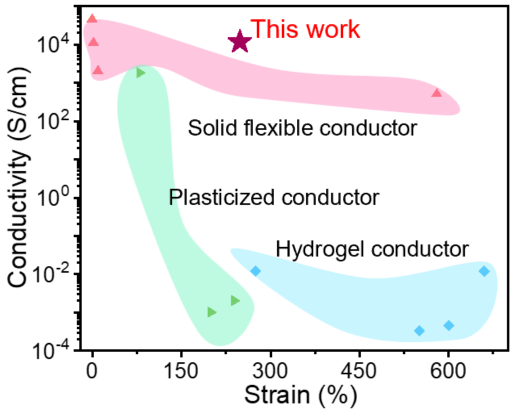

Supplementary Figure 10 Comparison of the tensile properties and conductivity of this composite conductive material pPEAOI-Ag-70 with different categories of stretchable conductive materials[24-34].

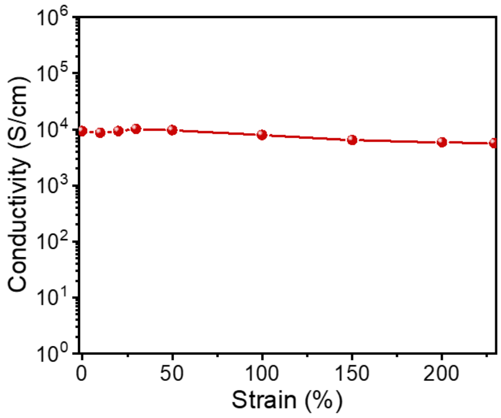

Supplementary Figure 11 Conductivity-strain characteristics of nanocomposite conductor pPEAOI-Ag-70.

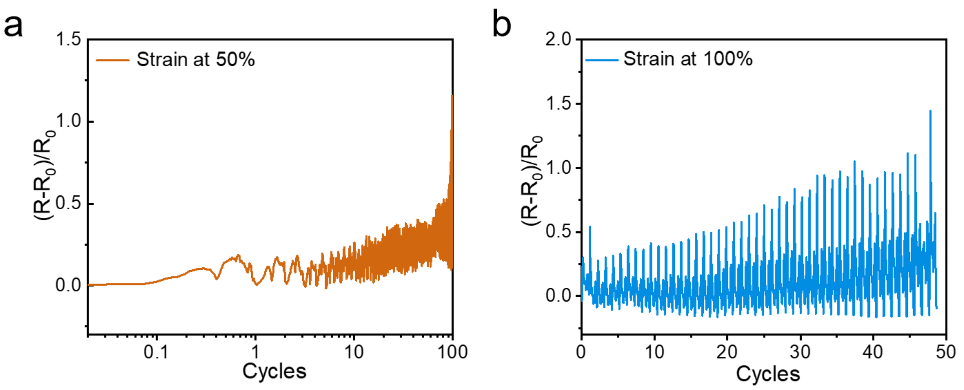

Supplementary Figure 12 The resistance changes of pPEAOI-Ag-70 during (a) 50% cyclic stretching and (b) 50% cyclic stretching.

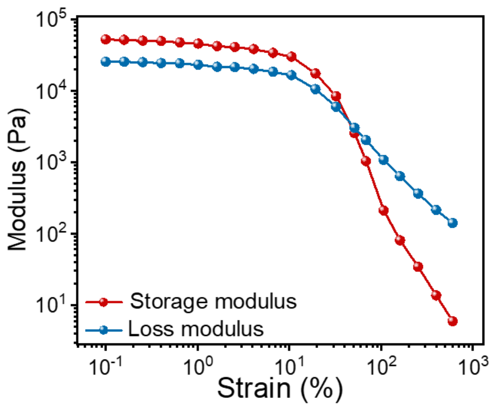

Supplementary Figure 13 Oscillation-amplitude sweep curve of pPEAOI-Ag-70 at 1 Hz, 25 °C.

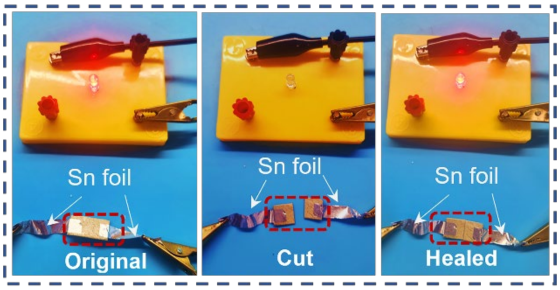

Supplementary Figure 14 The small bulb was utilized as a reference to demonstrate the instantaneous self-healing of electrical properties.

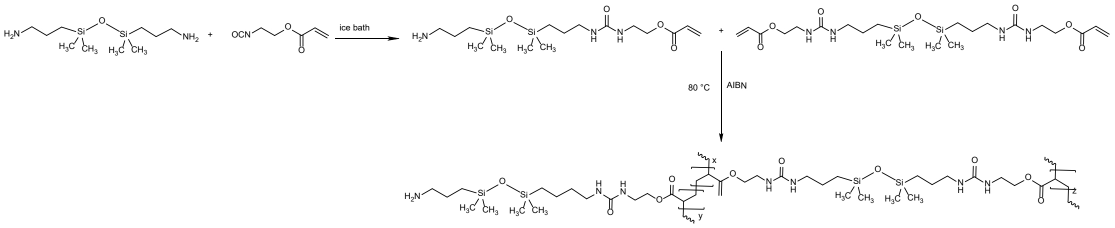

Supplementary Figure 15 The synthetic route of polymer binder pDMSOI.

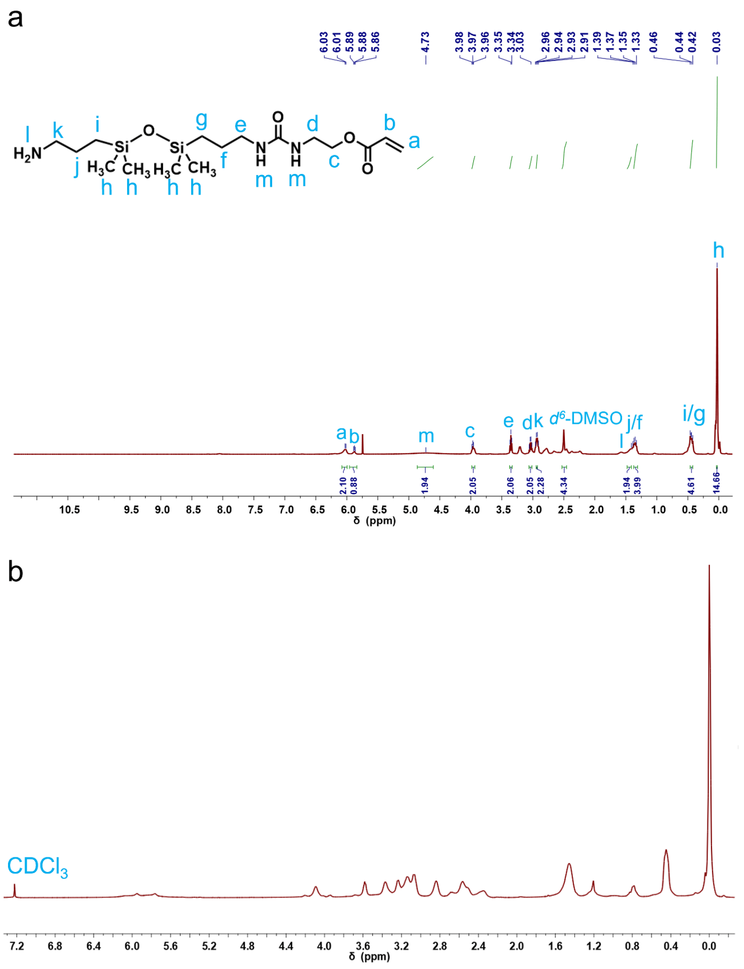

Supplementary Figure 16 ^{1}H NMR spectrum of (a) DMSOI: ^{1}H NMR (400 MHz, d^6-DMSO): δ (ppm) 6.02 (d, J = 5.2 Hz, 2H), 5.88 (t, J = 5.3 Hz 1H), 4.73 (s, 2H), 3.97 (m, 2H), 3.35 (t, J = 5.8 Hz, 2H), 3.03 (m, 2H), 2.93 (m, 2H), 1.41 (s, 2H), 1.35 (m, 4H), 0.44 (m, 4H), 0.03 (s, 12H). and (b) pDSMOI in $CDCl_3$.

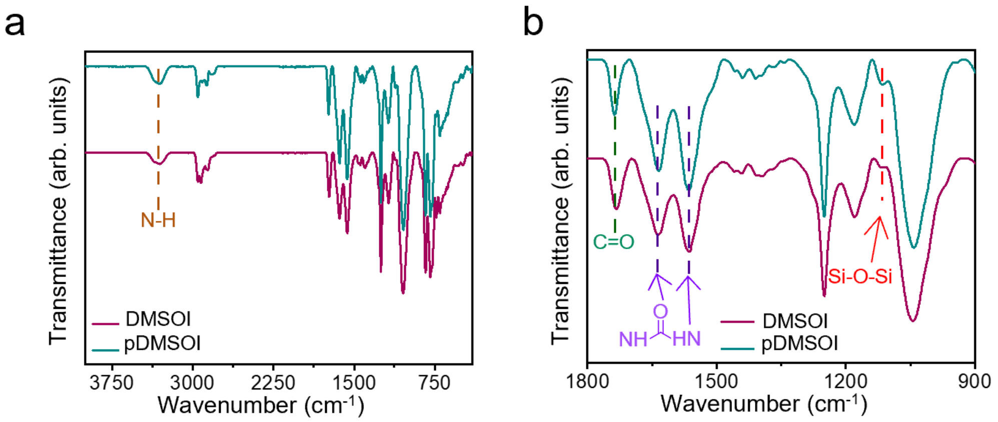

Supplementary Figure 17 Fourier transform infrared spectroscopy (FT-IR) of DMSOI and pDMSOI from (a) 4000 to 400 cm^{-1} and (b) partial enlargement from 1800 to 900 cm^{-1}.

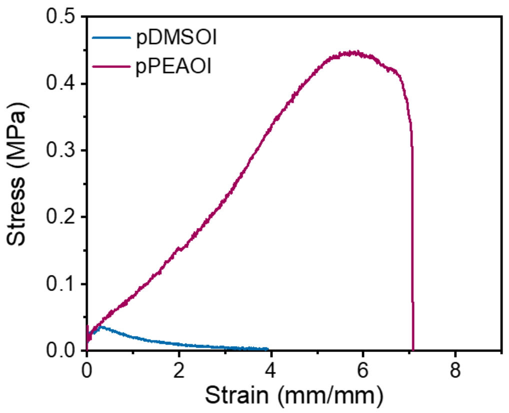

Supplementary Figure 18 Stress-strain curves of pDMSOI and pPEAOI.

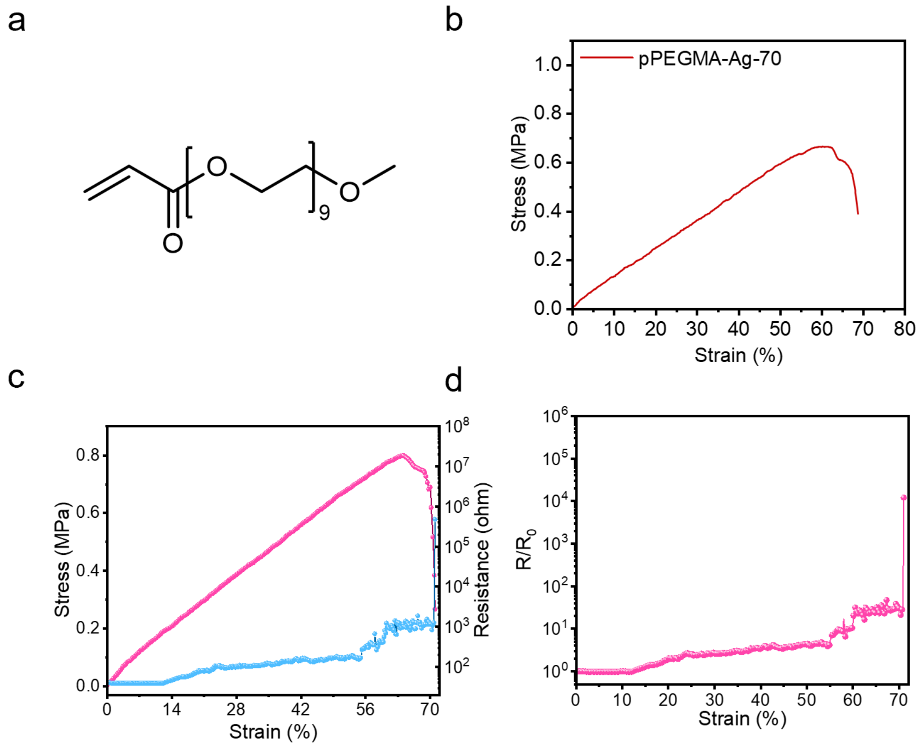

Supplementary Figure 19 pPEGMA which only has ether chains without urea bonds as a control polymer binder to prove the positive effect of heteroatoms-rich polymer binder. (a) Chemical structure of PEGMA. (b) Stress-strain curves of pPEGMA-Ag-70 under speed of deformation at 10 mm/min. (c) Resistance change and (d) relatively resistance value (R/R_0) change varies with strain during stretching.

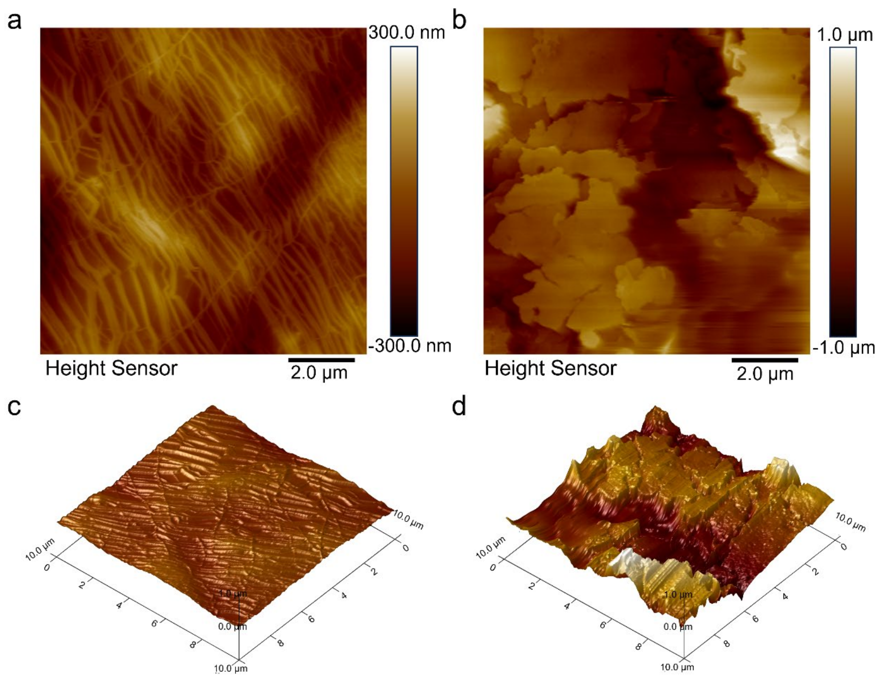

Supplementary Figure 20 Atomic Force Microscope (AFM) images of (a) nanocomposite conductor in this work and (b) pPEGMA-Ag-70. AFM (Atomic Force Microscope) images of (c) nanocomposite conductor in this work (*Ra* = 40.6 nm) and (d) pPEGMA-Ag-70 (*Ra* = 186.0 nm). The test area is 1 μm × 1 μm.

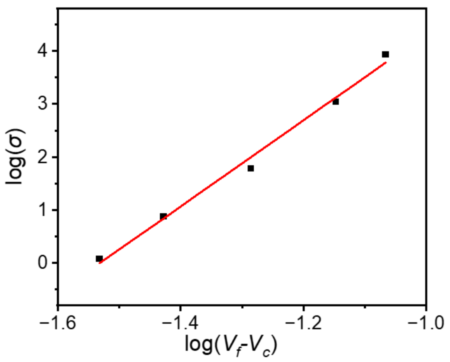

Supplementary Figure 21 Experimental dependence of σ on V_f and its linearization according to the classical power-law relation of 3D percolation theory[35]. Log plots of σ in respect to log(V_f–V_c) with a linear fit. The classical power law relates the conductivity of an unstretched material σ to V_f as follows:

$$\sigma = \sigma_0(V_f - V_c)^s$$

where σ_0 is the conductivity of the filler, V_f, and V_c are the volumetric fractions of the filler and percolation threshold at initial state, respectively, and s is the critical exponent[35]. To facilitate fitting, the equation was subjected to the following logarithmic transformation.

$$\log\sigma = \log\sigma_0 + s\log(V_f - V_c)$$

The lowest possible percolation threshold of nanocomposite conductor in this work was determined to be 11.4% volume fraction when the experimental data exhibited a perfect linear fit based on the equation (R^2 = 0.9917).

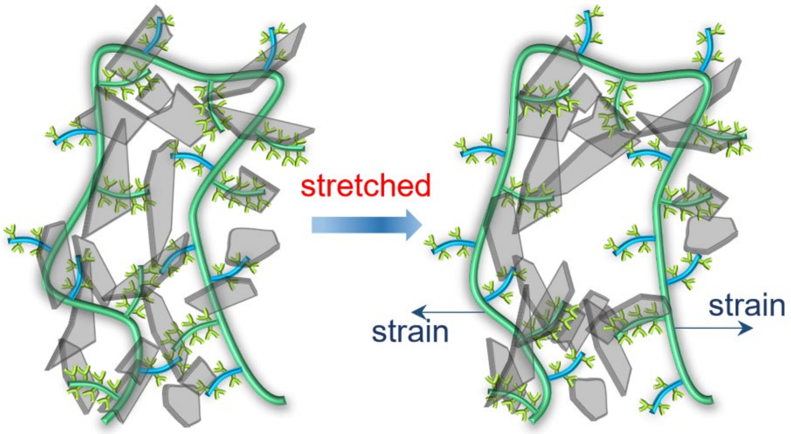

Supplementary Figure 22 The mechanism of energy dissipation and stable interconnected conductive road of nanocomposite pPEAOI-DMSOI-y-Ag-70 with mixed polymer binder.

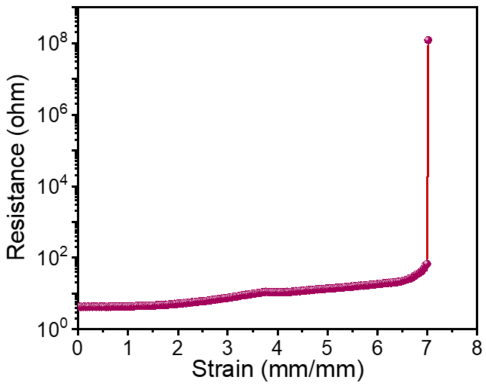

Supplementary Figure 23 Resistance change of pPEAOI-DMSOI-50-Ag-70 as a function of uniaxial strain at 100 mm/min.

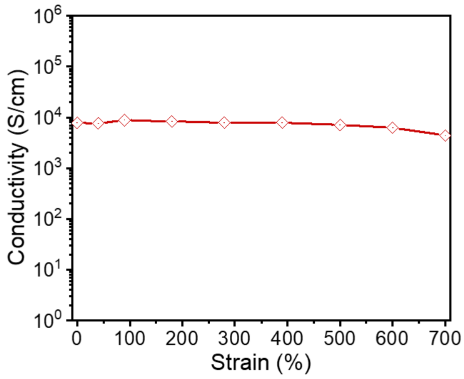

Supplementary Figure 24 Conductivity-strain characteristics of nanocomposite conductor pPEAOI-DMSOI-50-Ag-70.

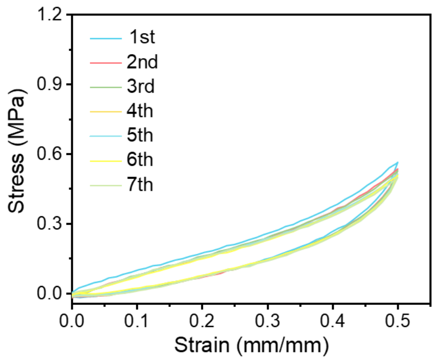

Supplementary Figure 25 Curves of pPEAOI-DMSOI-50-Ag-70 stretched and relaxed under 50% strain at deformation speed of 100 mm/min.

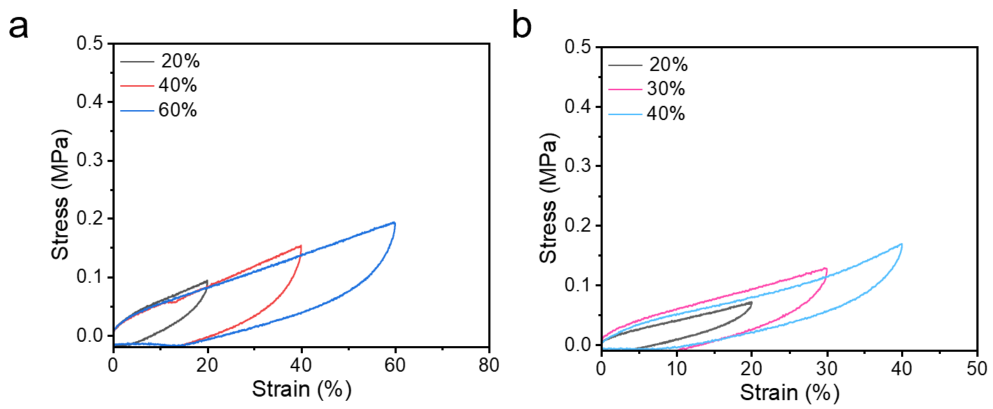

Supplementary Figure 26 Curves of pPEAOI-DMSOI-50-Ag-70 stretched and relaxed under different strains at deformation speed of (a) 2 mm/min and (b) 3 mm/min.

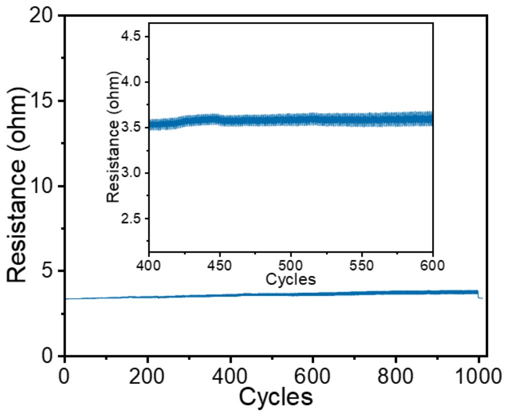

Supplementary Figure 27 Change in resistance of pPEAOI-DMSOI-50-Ag-70 over 1000 stretch-relaxation cycles to 50% strain, the small figure is a zoomed-in view of 400-600 cycles .

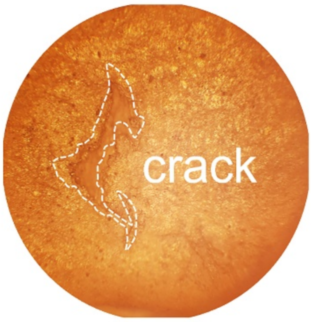

Supplementary Figure 28 Optical microscope image of pPEAOI-DMSOI-50-Ag-70 after 1000 stretch-relaxation cycles to 50% strain.

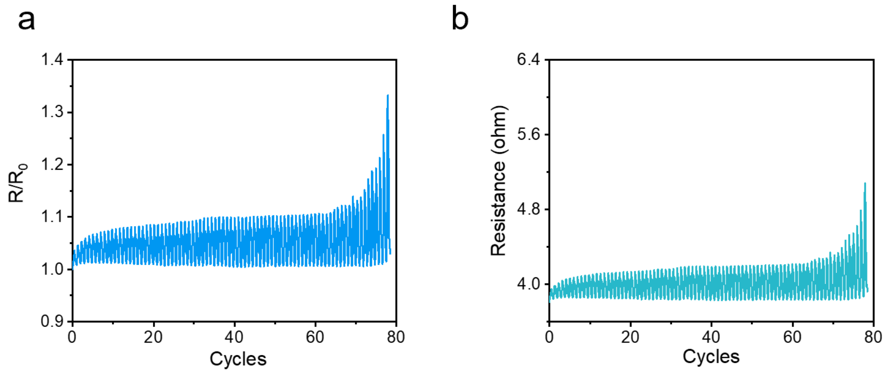

Supplementary Figure 29 Change in (a) relative resistance and (b) resistance of pPEAOI-DMSOI-50-Ag-70 over 78 stretch-relaxation cycles to 100% strain.

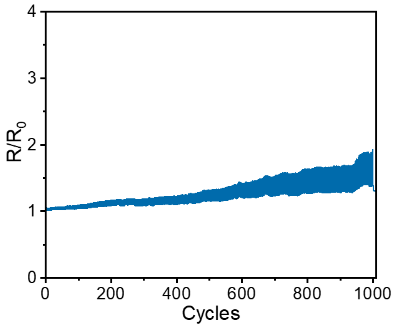

Supplementary Figure 30 Change in relative resistance of pPEAOI-DMSOI-50-Ag-70-cold pressing over 1000 stretch-relaxation cycles to 50% strain.

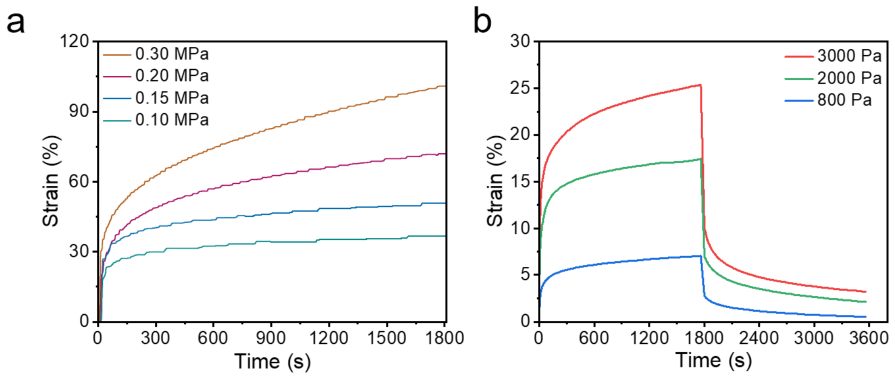

Supplementary Figure 31 Creep characterization of pPEAOI-DMSOI-50-Ag-70. (a) Creep strain varies with time under different tensile stress. Under uniaxial constant stresses of approximately 0.10 MPa, 0.15 MPa, 0.20 MPa, and 0.30 MPa for 30 minutes at room temperature, the creep strain of the nanocomposite conductor pPEAOI-DMSOI-50-Ag-70 reached 13.20%, 19.91%, 42.27%, and 58.29%, respectively. (b) Curves of creep and recovery under different shear stress at 25 ˚C. After the shear stress was released and the sample was allowed to recover for 30 minutes, the strain decreased from 7.03% to 0.52%, from 17.43% to 2.12%, and from 25.35% to 3.21%, respectively.

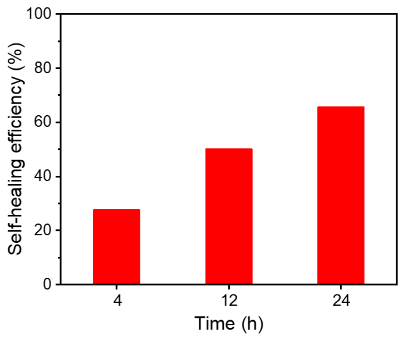

Supplementary Figure 32 Self-healing efficiency of pPEAOI-DMSOI-50-Ag-70 repaired at ambient temperature for different periods.

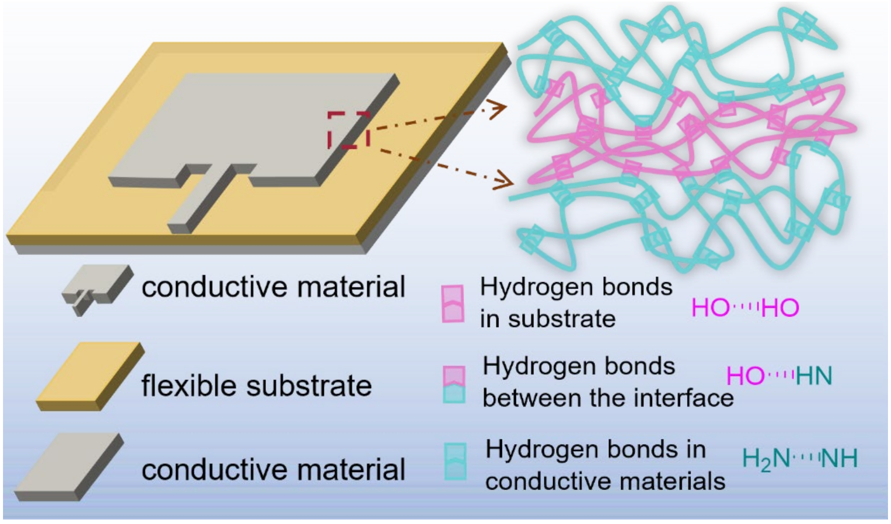

Supplementary Figure 33 The interaction at the interface of the flexible antenna.

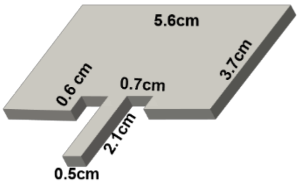

Supplementary Figure 34 The parameters of the metallic pattern.

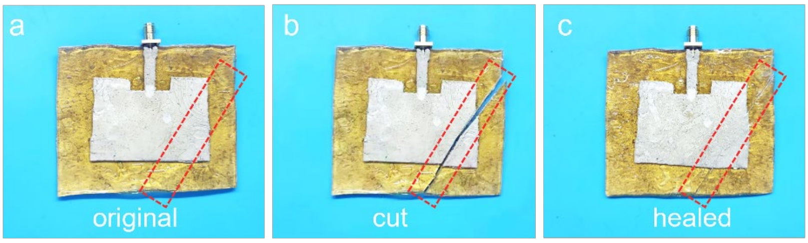

Supplementary Figure 35 Optical images of the flexible antenna during the self-healing process (a) original state; (b) cut state and (c) repaired state.

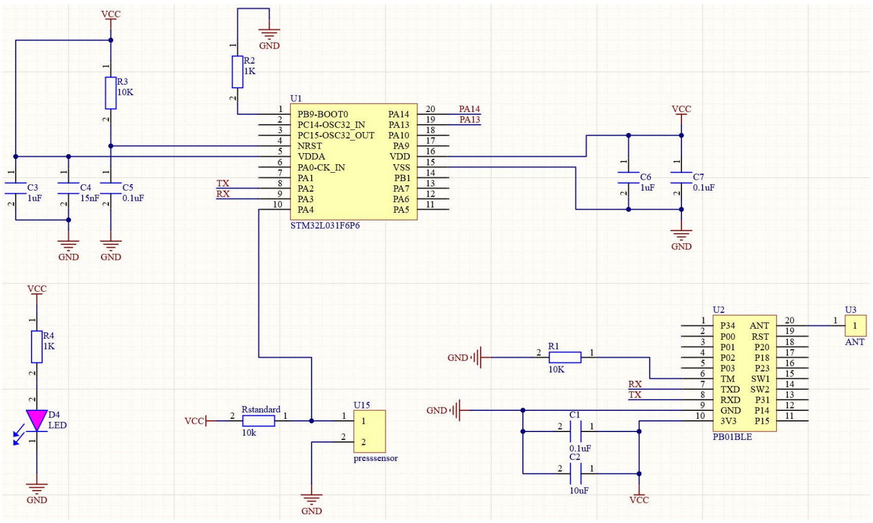

Supplementary Figure 36 The schematic diagram of the wireless touch-interaction system.

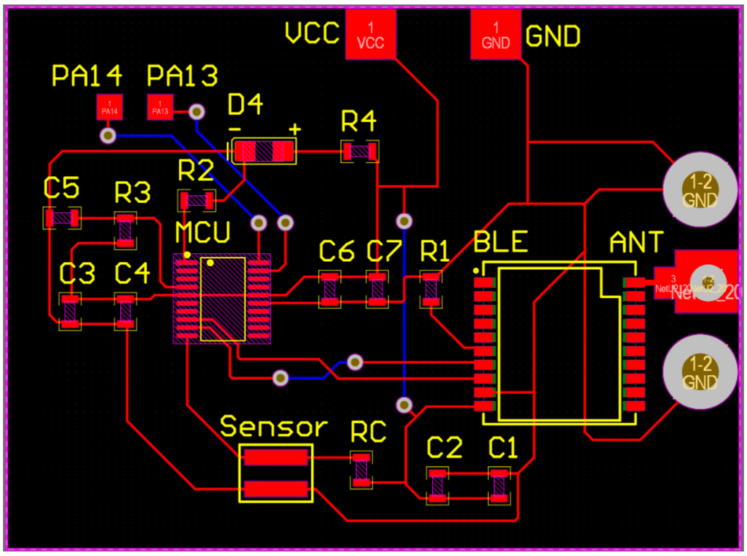

Supplementary Figure 37 The PCB layout of the wireless touch-interaction system.

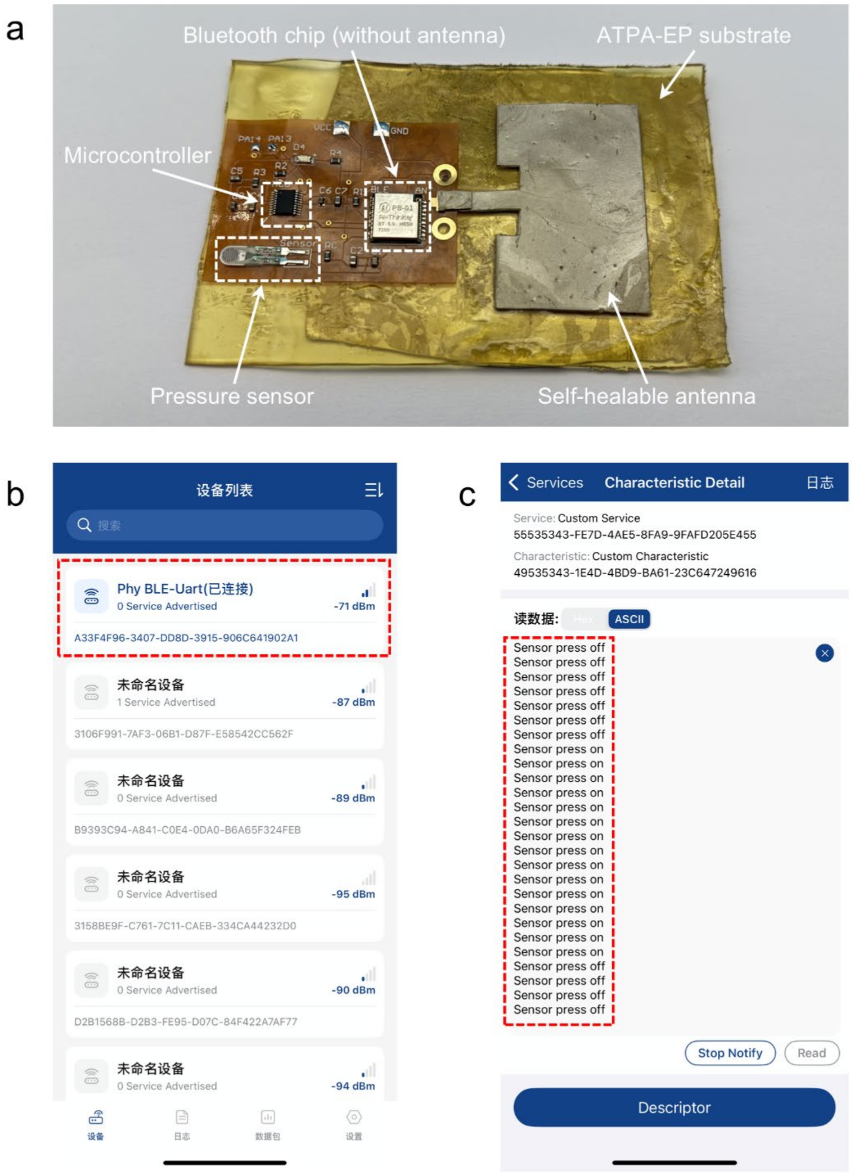

Supplementary Figure 38 The wireless touch-interaction system and wireless communication demonstration. (a) The photo of the fabricated wireless touch-interaction system. (b) The connection status when the distance between mobile phone and wireless system is 70 m. (c) The data transmission of the touch-interaction system when the communication distance is 70 m.

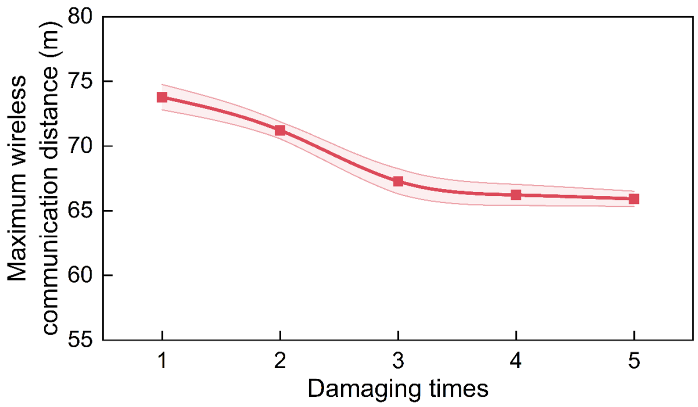

Supplementary Figure 39 Maximum wireless communication distance of the wearable sensing system after several times of damaging experiments. The damage-resilience experiment was carried out by repeatedly cutting the central region of the antenna's radiation structure.

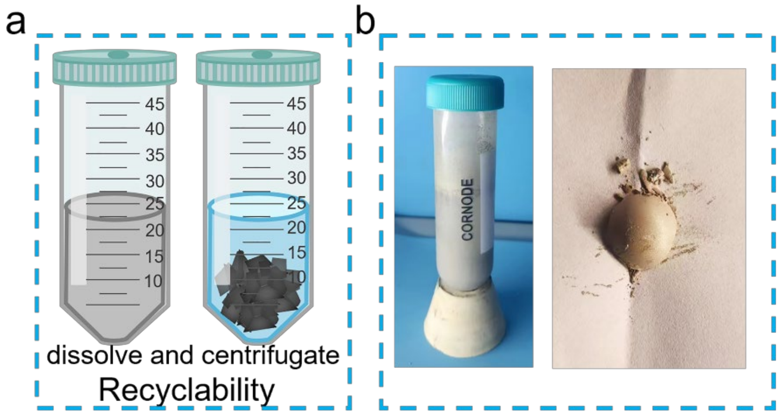

Supplementary Figure 40 Recycle Ag flakes from the flexible antenna. (a) Schematic diagram and (b) optical images of Ag flakes recycling.

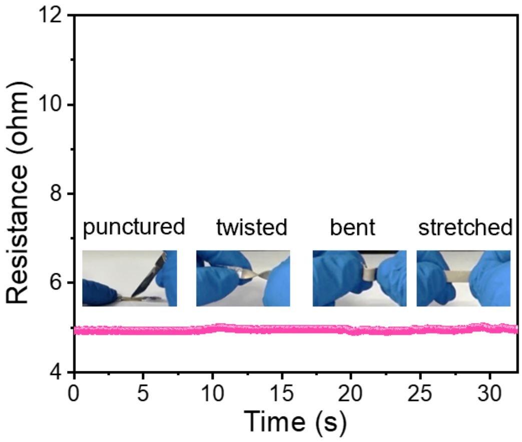

Supplementary Figure 41 Real-time resistance of nanocomposite conductor made from recycled Ag flakes under mechanical damage or deformation.

Supplementary Tables

Supplementary Table 1 Comparisons of pPEAOI-DMSOI-50-Ag-70 with various stretchable conductors recently reported.

**Reference**	**Polymer binder**	**Conductive filler**	**Conductivity (S/cm)**	**Flexibility**	**Strain-insensitivity**	**Repairing ability**	**Durability**
Nat. Nanotechnol.[1]	Porous PU	Ag NWs	8152	750%	$R/R_0 = 20.2$ Strain at 500%	No	10000 cycles
Nat. Nanotechnol.[2]	SEBS	Au-Ag NWs	41850	266%	$R/R_0>10$ Strain at 100%	No	N.A.
Science[3]	SEBS	Ag NWs	>100000	1000%	$R/R_0=100$ Strain at 500%	No	N.A.
Nat. Electron.[4]	PDMS	Au	6.33×10^{-7} Ω m	350%	$R/R_0 = 6$ Strain at 100%	No	5000 cycles
Adv. Mater.[5]	PU	Ag NWs	9190	310	$R/R_0 =1.4$ Strain at 50%	No	1000 cycles
Nature[6]	PU	Ag NPs	11000	115	$R/R_0>10$ Strain at 100%	No	10000 cycles
Adv. Mater.[7]	SEBS	Ag NPs	31000	800%	$R/R_0 = 1.01$ Strain at 50%	No	1000 cycles
Nat. Mater.[8]	Fluorine rubber	Ag flakes	6168	410%	$R/R_0 = 1.01$ Strain at 50%	No	1000 cycles
Nat. Electron.[9]	PAAm-Alginate hydrogel	Ag flakes	374	250%	$R/R_0 = 30$ Strain at 200%	No	1000 cycles
Nat. Nanotechnol.[10]	Supramolecular	μNi particle	40	20%	N.A.	Yes	N.A.
Nat. Commun.[11]	VegPU	Ag flakes	12833	350%	N.A.	No	1000 cycles
Nat. Commun.[12]	AATN hydrogel	Ag NWs	58	1230%	$R/R_0 = 1.2$ Strain at 500%	Yes	500 cycles
Adv. Mater.[13]	PEG/PENI/PTA	Ag flakes	60000	1000%	$R/R_0 < 2$ Strain at 100%	No	1000 cycles
Challenges (**Metallic composites**)	✘ Limited electronic conductivity<br>✘ Self-healing ability<br>✘ Low strain-insensitivity<br>✘ Stability of substrate (especially for hydrogel)						
PNAS[14]	SIS	Cu/liquid metal	120000	1200%	$R/R_0 = 3$ Strain at 300%	Electrically self-healing	1000 cycles
Nat. Mater.[15]	TPU	Liquid metal	2130000	420%	$R/R_0 = 1.036$ Strain at 100%	No	10000

Nat. Mater.[16]	SBS	Liquid metal	1800000	2300%	$R/R_0 = 1$ Strain at 500%	No	1000 cycles
Sci. Adv.[17]	PVDF-HFP-TFE	Liquid metal	43500	1170	$R/R_0 = 1.04$ Strain at 200%	No	N.A.
Challenges (**LM composites**)	× Self-healing ability<br>× Leaking of LM or corrosion with metal components						
Nat. Commun.[18]	PDMS	SWCNT	> 0.02	400%	$R/R_0 = 1.25$ Strain at 100%	No	1000 cycles
Sci. Adv.[19]	polysilicone	Graphene	0.001	100%	$R/R_0 = 1.03$ Strain at 1%	No	N.A.
J. Mater. Chem. C[20]	Silicone rubber	Graphene/ Ag NF	200	140%	$R/R_0 = 1.25$ Strain at 30%	No	N.A.
J. Mater. Chem. C[21]	TPU	Graphene	0.0001	300%	N.A.	No	N.A.
Nat. Commun.[22]	PDA	Ti_3C_2	0.16Ω sq^{-1}	N.A.	N.A.	No	N.A.
ACS Nano[23]	PAA-b-PBA	PANI	100	90%	N.A.	No	N.A.
Challenges **(carbon-based/other composites)**	× Self-healing ability<br>× Limited flexibility or easy to breakage<br>× Low electronic conductivity<br>× Lack of strain resilience						
**This work**	pPEAOI-DMSOI	Ag flakes	9382	720%	$R/R_0 = 15.85$ Strain at 700%	Yes	1000 cycles
	✓ Simple one-pot preparation process<br>✓ Excellent electrical conductivity<br>✓ High stretchability exceeding 700%<br>✓ Strain-insensitive electrical performance ($R/R_0 = 1.22$, Strain at 200%)<br>✓ Superior durability and ultralow electrical hysteresis against cyclic stretching tests						

Supplementary References

1. Xu, Y. *et al.* Phase-separated porous nanocomposite with ultralow percolation threshold for wireless bioelectronics. *Nature Nanotechnology*, **19**, 1158-1167 (2024).
2. Choi, S. *et al.* Highly conductive, stretchable and biocompatible Ag-Au core–sheath nanowire composite for wearable and implantable bioelectronics. *Nature Nanotechnology* **13**, 1048-1056 (2018).
3. Jung, D. *et al.* Highly conductive and elastic nanomembrane for skin electronics. *Science* **373**, 1022-1026 (2021).
4. Jiang, Z. *et al.* A 1.3-micrometre-thick elastic conductor for seamless on-skin and implantable sensors. *Nature Electronics* **5**, 784-793 (2022).
5. Jiang, Z. *et al.* Highly stretchable metallic nanowire networks reinforced by the underlying randomly distributed elastic polymer nanofibers via interfacial adhesion improvement. *Advanced Materials* **31**, 1903446 (2019).
6. Kim, Y. *et al.* Stretchable nanoparticle conductors with self-organized conductive pathways. *Nature* **500**, 59-63 (2013).
7. Jung, D. *et al.* Adaptive self-organization of nanomaterials enables strain-insensitive resistance of stretchable metallic nanocomposites. *Advanced Materials* **34**, 2200980 (2022).
8. Matsuhisa, N. *et al.* Printable elastic conductors by in situ formation of silver nanoparticles from silver flakes. *Nature Materials* **16**, 834-840 (2017).
9. Ohm, Y., Pan, C., Ford, M. J., Huang, X., Liao, J. & Majidi, C. An electrically

conductive silver-polyacrylamide-alginate hydrogel composite for soft electronics. *Nature Electronics* **4**, 185-192 (2021).

10. Tee, B. C. K., Wang, C., Allen, R. & Bao, Z. An electrically and mechanically self-healing composite with pressure- and flexion-sensitive properties for electronic skin applications. *Nature Nanotechnology* **7**, 825-832 (2012).

11. Lv, J. *et al.* Printed sustainable elastomeric conductor for soft electronics. *Nature Communications* **14**, 7132 (2023).

12. Song, P., Qin, H., Gao, H.-L., Cong, H.-P. & Yu, S.-H. Self-healing and superstretchable conductors from hierarchical nanowire assemblies. *Nature Communications* **9**, 2786 (2018).

13. Wang, T., Liu, Q., Liu, H., Xu, B. & Xu, H. Printable and highly stretchable viscoelastic conductors with kinematically reconstructed conductive pathways. *Advanced Materials* **34**, 2202418 (2022).

14. Li, Y. *et al.* Ultrasensitive and ultrastretchable electrically self-healing conductors. *Proceedings of the National Academy of Sciences* **120**, e2300953120 (2023).

15. Zheng, S., Wang, X., Li, W., Liu, Z., Li, Q. & Yan, F. Pressure-stamped stretchable electronics using a nanofibre membrane containing semi-embedded liquid metal particles. *Nature Electronics* **7**, 576-585 (2024).

16. Ma, Z. *et al.* Permeable superelastic liquid-metal fibre mat enables biocompatible and monolithic stretchable electronics. *Nature Materials* **20**, 859-868 (2021).

17. Zheng, L., Zhu, M., Wu, B., Li, Z., Sun, S. & Wu, P. Conductance-stable liquid metal sheath-core microfibers for stretchy smart fabrics and self-powered sensing. *Science Advances* **7**, eabg4041 (2021).

18. Xu, P. *et al.* Conductive and elastic bottlebrush elastomers for ultrasoft electronics. *Nature Communications* **14**, 623 (2023).

19. Boland, C. S. *et al.* Sensitive electromechanicalsensors using viscoelasticgraphene-polymer nanocomposites. *Nature Communications* **14**, 623 (2023).

20. Wang, Y., Zhu, L., Mei, D. & Zhu, W. A highly flexible tactile sensor with an interlocked truncated sawtooth structure based on stretchable graphene/silver/silicone rubber composites. *Journal of Materials Chemistry C* **7**, 8669-8679 (2019).

21. Liu, H. *et al.* Electrically conductive thermoplastic elastomer nanocomposites at ultralow graphene loading levels for strain sensor applications. *Journal of Materials Chemistry C* **4**, 157-166 (2016).

22. Zhao, W. *et al.* 2D Titanium carbide printed flexible ultrawideband monopole antenna for wireless communications. *Nature Communications* **14**, 278 (2023).

23. Ding, H. *et al.* Elastomeric conducting polyaniline formed through topological control of molecular templates. *ACS Nano* **10**, 5991-5998 (2016).

24. Ma, R. & Tsukruk, V. V. Seriography-guided reduction of graphene oxide biopapers for wearable sensory electronics. *Advanced Functiional Materials* **27**, 1604802 (2017).

25. Lian, M. *et al.* Gelatin-assisted fabrication of graphene-based nacre with high strength, toughness, and electrical conductivity. *Carbon* **89**,279-289 (2015).

26. Lee, T. H., Kim, J. H. & Lee, J. Y. Fabrication of highly conductive fibers bymetal ion-exchange using a simply modified wet-spinning process. *Macromolecular Research* **25**,1230-1236 (2017).

27. Han,W. B. *et al.*Ultra-stretchableandbiodegradableelastomersfor soft, transient electronics. *Nature Communications* **14**, 2263 (2023).

28. Ling, S. *et al.* Integration of stiff graphene and tough silk for the design and fabrication of versatile electronic materials. *Advanced Functional Materials* **28**, 1705291 (2018).

29. López Barreiro, D. *et al.* Conductive silk-based composites using biobased carbon materials. *Advanced Materials* **31**, 1904720 (2019).

30. Yan, L. *et al.* Conductive cellulose bio-nanosheets assembled bio stable hydrogel for reliable bioelectronics. *Advanced Functional Materials* **31**, 2010465 (2021).

31. Song, J. *et al.* Mechanically and electronically robust transparent organohydrogel fibers. *Advanced Materials* **32**, 1906994 (2020).

32. Ye, Y., Zhang, Y., Chen, Y., Han, X. & Jiang, F. Cellulose nanofibrils enhanced, strong, stretchable, freezing-tolerant ionic conductive organohydrogelformulti-functional sensors. *Advanced Functional Materials* **30**, 2003430 (2020).

33. Yang, J. *et al.* Antifreezing Zwitterionic Hydrogel Electrolyte with High Conductivity of 12.6 mS cm^{-1} at −40 °C through Hydrated Lithium Ion Hopping

Migration. *Advanced Functional Materials* **31**, 2009428 (2021).

34. Bai, Y. *et al.* A Stretchable Polymer Conductor Through the Mutual Plasticization Effect. *Advanced Materials* **35**, 2303245 (2023).

35. Li, J. & Kim, J.-K. Percolation threshold of conducting polymer composites containing 3D randomly distributed graphite nanoplatelets. *Composites Science and Technology* **67**, 2114-2120 (2007).
