# Supplementary material for: Strain resilient and self-healing nanocomposite conductors with ultralow sheet resistance
Source: Nat Commun. 2026 May 12;17:6344. doi: 10.1038/s41467-026-71851-9 (PMC13376812; doi:10.1038/s41467-026-71851-9)
Supplement: Supplementary file 2 — Description Of Additional Supplementary File [file 41467_2026_71851_MOESM2_ESM.pdf]

Description of Additional supplementary files

Supplementary Movie 1 The stretchability and relaxation behavior of pPEAOI-DMSOI-50-Ag-70.

Supplementary Movie 2 The ultrastable conductivity of the nanocomposite conductor under puncture and deformation conditions.

Supplementary Movie 3 The wireless communication demonstration of the wearable sensing system based self-healing nanocomposite conductors.
