# Supplementary material for: Strain resilient and self-healing nanocomposite conductors with ultralow sheet resistance
Source: Nat Commun. 2026 May 12;17:6344. doi: 10.1038/s41467-026-71851-9 (PMC13376812; doi:10.1038/s41467-026-71851-9)
Supplement: Supplementary file 6 — Transparent Peer Review file [file 41467_2026_71851_MOESM6_ESM.pdf]

nature portfolio

Peer Review File

Strain Resilient and Self-healing Nanocomposite Conductors with Ultralow Sheet Resistance

Corresponding Author: Professor Cheng-Hui Li

This file contains all reviewer reports in order by version, followed by all author rebuttals in order by version.

Version 0:

Reviewer comments:

Reviewer #1

(Remarks to the Author)
The paper "Strain Resilient and Self-healing Nanocomposite Conductors with Ultralow Sheet Resistance" reports self-healable Ag flake/polymer binder composites that can be stretched without sacrificing resistance. This work suggested brush polymer binder which can promote the directional distribution of conductive fillers. This is an exciting and promising concept.However, based on the contents described in this manuscript, the necessity of this concept remains unclear. There should be some more data that can support the author's claim. Thus, I think this manuscript can be accepted after some revision.
[1] Although hot pressing is the key factor that induces directional distribution, this processing method is commonly employed in most composite studies. Is there any specific evidence suggesting that the binder itself can further promote such directional distribution?

[2] There is no direct evidence confirming the presence of "coordination interaction" in which the lone pairs of the polymer binder stabilize the Ag flakes. If the authors intend to claim the presence of coordination interactions, they should additionally provide supporting data (e.g., FT-IR, XPS, Raman spectroscopy) along with the DSC curves in Fig. 2e.

[3] The claim that a directional distribution is achievable seems to imply that the percolation threshold can be reduced. I am curious about the minimum filler concentration at which the percolation threshold is reached using this approach, and whether it can be significantly lowered compared to other systems.

[4] In addition, it would be helpful to present the conductivity or sheet resistance depend on weight percent of Ag flake.

[5] In the mechanism section, additional comparisons with appropriate controls are needed—not as plasticizers for self-healing, but as systems that can help elucidate the underlying mechanism. This paper presents a siloxane-based control; however, siloxane is well known for its low chain friction and low Tg. Therefore, the observed low stress level and excellent self-healing performance are rather expected results. If the authors intend to emphasize the effect of the brush polymer binder possessing abundant lone pairs, it is essential to include a comparison with another system containing ether chains but fewer lone pairs (e.g., PEGDMA).

Reviewer #2

(Remarks to the Author)
Hou et al. presents a stretchable and self-healing conductor for stretchable electronics by introducing conductive fillers into a bottlebrush polymer binder. In this creeper-plant-inspired architecture, the conductive fillers (leaves) provide low resistance, whereas the polymer binders (branches) impart stretchability and self-healing capability. This design enabled the flexible conductor to maintain high conductivities (11574 S/cm) under repeated strain cycles (50 and 100%). Furthermore, hydrogen bonding within the bottlebrush chains allowed the conductors to self-heal after damage without a major loss in conductivity. The optimization of the heteroatom content in the bottlebrush to enhance the strain and fatigue resistance of the material while retaining self-healing properties was a nice experiment. The authors note that there is a trade-off between "self-healing capability, conductivity, and flexible deformability of nanocomposite conductors". Can the authors explain why there is trade-

off between self-healing capability and flexibly deformability? My impression is that if something is easy to deform, it will be easier to “self-heal” as well. As well documented by the authors, there are many examples of self-healing conductors. My major critique of these work is that while some mechanical properties are improved, there are important mechanical properties that are lost. For instance, often the elongation at break are high and this work similarly shows this is true as well. However, for many materials that heal well, the elasticity is poor. It is very important to show the cycling data at different stain percentages to show there is hysteresis (or lack there of) at different strain rates. Otherwise, you can have a very stretchable material but cannot return back to its original shape. Indeed the authors show cycling data of the samples (such as figure 3d/e, it would be helpful to show real images rather than a cartoon here), but this is showing how the resistance changes rather than showing the mechanical properties. There is partial data (S16), but there is only a handful of cycles an an undefined strain rate. It is important to show the slower strain rates as this is where viscous properties are elucidated. Since the authors show some stretching videos, it would nice to show how the material relaxes. Additionally, creep data is highly recommended as this paper should include full mechanical characterization data—this is easily collected on the same instruments. For me, this data is necessary for publication in Nature Communications. For proper publications, please include the ppm shifts, integration, and j-couplings for small molecules in the NMR (SI). Lastly, I am unsure about the point about recyclable Ag flakes, as it is brought up at the end and it is not well motivated in the story of the manuscript. If it is included, then it would be nice to show that the flakes can be reintroduced into a new composite with equivalent electrical properties under mechanical deformation.

Reviewer #3

(Remarks to the Author)
This manuscript reports the design of a creeper-inspired, self-healable and highly stretchable conductive nanocomposite based on Ag flakes embedded in a viscoelastic brush polymer binder. By hot pressing, the authors induce a surface-enriched, multilayer distribution of Ag flakes, achieving an ultralow initial sheet resistance (~10.8 mΩ sq^{-1}) and high conductivity. The composite exhibits large mechanical stretchability (up to ~700%), partial mechanical self-healing, and recovery of electrical continuity after damage. Leveraging these properties, the authors fabricate a stretchable, self-healable antenna operating at 2.4 GHz and demonstrate a wireless e-skin system capable of Bluetooth communication over tens of meters under small mechanical deformation.

Although the experimental effort is substantial, the work suffers from fundamental shortcomings in performance benchmarking, terminology accuracy, and application relevance. Moreover, the emphasis on bio-inspired design is overstated – there is only superficial similarity between the material microstructure and the Virginia Creeper plant and not enough scientific support for more fundamental commonalities between the two. While I think that this paper is a potentially valuable contribution to the field, the authors should address the following issues for this work to merit publication:

1. The creeper-inspired design with directionally accumulated distribution of Ag flakes is presented as a key concept. While SEM and EDS data confirm surface-enriched filler distribution after hot pressing, the manuscript does not provide a direct comparison between this structure and a randomly distributed Ag-flake composite with otherwise similar composition. To fully support the claimed advantage of this directional architecture, it would be valuable to demonstrate how it improves electrical stability, hysteresis, or durability relative to a control sample without induced directional migration. Without such comparison, the benefits of the creeper-inspired design remain unrevealed.

2. The authors report that for “pPEAOI-DMSOI-50-Ag-70”, the electrical resistance increases to approximately “64 Ω at 700 % strain” (as shown in Fig. 4e and Fig. S15). While the corresponding normalized resistance change ($R/R_0 \approx 15.8$) is lower than many previously reported stretchable conductors, the absolute resistance value is still relatively high. Such resistance levels seem non-trivial for practical applications including RF interconnects requiring low noise and minimal RC delay. Therefore, it would be helpful if the authors could clarify the operational strain condition for practical electronic and RF applications and distinguish it from the extreme-strain mechanical survivability demonstrated in tensile tests.

3. The term “strain-insensitive conductivity” is used repeatedly throughout the manuscript. However, the experimental data indicate that:
• At “200 % strain, $R/R_0 \approx 1.22$,” which is reasonably supports this description.
• At “700 % strain, $R/R_0 \approx 15.8$,” representing a significant resistance increase.
Although this behavior is still impressive relative to prior work, describing the electrical conductivity of the composite as “strain-insensitive across the entire strain range” appears a little bit misleading. The authors are encouraged to clearly define the strain range over which the material can be considered electrically “strain-insensitive”.

4. Reporting on sample geometry and conductivity measurement under strain are unclear. While resistance–strain curves are shown, the manuscript does not clearly specify the sample geometry and dimensions used for resistance measurements. Because geometric elongation alone can significantly affect resistance, it would be important to clarify how these factors were accounted for. Providing electrical conductivity or resistivity data under strain, or explicitly stating measurement assumptions, would strengthen the quantitative interpretation.

5. The presentation of the supplementary movies is not sufficiently quantitative or informative. While the videos qualitatively illustrate stretchability and electrical continuity, they lack clear annotations of the data. Without these elements, the supplementary movies do not meaningfully support the quantitative claims made in the manuscript and should be improved to provide scientifically rigorous, data-related visualization.

Version 1:

Reviewer comments:

Reviewer #1

(Remarks to the Author)
Authors have addressed my comments appropriately. I am satisfied with the revisions and recommend this work for publication in Nature Communications.

Reviewer #2

(Remarks to the Author)
My comments are adequately addressed; however, after reading the other reviewers comment, I must also say I think the analogy to the plant is a stretch. I recommend it is removed. I almost made the same comment but was on the fence.

Reviewer #3

(Remarks to the Author)
The authors have addressed all my previous concerns through additional experiments and detailed clarifications. The revised manuscript is significantly strengthened and now I think this meets the standards of Nature Communications. I recommend its publication.

Open Access This Peer Review File is licensed under a Creative Commons Attribution 4.0 International License, which permits use, sharing, adaptation, distribution and reproduction in any medium or format, as long as you give appropriate credit to the original author(s) and the source, provide a link to the Creative Commons license, and indicate if changes were made.
In cases where reviewers are anonymous, credit should be given to 'Anonymous Referee' and the source.
The images or other third party material in this Peer Review File are included in the article's Creative Commons license, unless indicated otherwise in a credit line to the material. If material is not included in the article's Creative Commons license and your intended use is not permitted by statutory regulation or exceeds the permitted use, you will need to obtain permission directly from the copyright holder.
To view a copy of this license, visit https://creativecommons.org/licenses/by/4.0/

Point-to-point response letter

REVIEWER COMMENTS

Reviewer #1 (Remarks to the Author):

The paper "Strain Resilient and Self-healing Nanocomposite Conductors with Ultralow Sheet Resistance" reports self-healable Ag flake/polymer binder composites that can be stretched without sacrificing resistance. This work suggested brush polymer binder which can promote the directional distribution of conductive fillers. This is an exciting and promising concept. However, based on the contents described in this manuscript, the necessity of this concept remains unclear. There should be some more data that can support the author's claim. Thus, I think this manuscript can be accepted after some revision.

Response: We appreciate your comments on our work. We have revised our manuscript thoroughly according to your suggestions, and all changes are highlighted in yellow in the revised manuscript.

[1] Although hot pressing is the key factor that induces directional distribution, this processing method is commonly employed in most composite studies. Is there any specific evidence suggesting that the binder itself can further promote such directional distribution?

Response: We sincerely appreciate your insightful comments. Indeed, hot-pressing is commonly employed in most composite studies. Many works reported that hot-pressing benefited to promote directional immigration of nanoparticles or flakes. To figure out the role of polymer binder, we try to find evidence using AFM (Atomic Force Microscope). Surprisingly, under the same Ag content (70%), hot-pressed nanocomposite conductors based on pPEAOI and pPEGMA with less heteroatoms

exhibit distinctly different surface morphologies. As shown in Figure S18a, Ag flakes on the surface of pPEAOI-Ag-70 show obviously orderly packed distribution which have significant positive effect to the continuous and stable conductive pathways, thus resulting in the ultralow sheet resistance. On the contrast, Ag flakes on the surface of pPEGMA-Ag-70 did not exhibit similar order distribution (Figure S18b). Therefore, we can propose a possible mechanism in which hot-pressing drive the immigration of Ag flakes from inside to the outside surface, at the same time, polymer binder composed of the nanocomposite conductor determines the order arrangement of the Ag flakes on the surface. We have revised Figure 1b to accurately represent the true morphology measured by AFM.

The relative discussion can be found on Page 14 in the revised manuscript as follows: "Atomic Force Microscope (AFM) was utilized to further investigate the role of polymer binders in the migration and oriented distribution of Ag flakes. As shown in Figure S18, Ag flakes on the surface of pPEAOI-Ag-70 show obviously orderly packed distribution and lower roughness ($Ra = 40.6$ nm), which have significant positive effects to the continuous and stable conductive pathways, thus resulting in ultralow sheet resistance and high conductivity. On the contrast, Ag flakes on the surface of pPEGMA-Ag-70 did not exhibit similar order distribution and pPEGMA-Ag-70 showed higher roughness ($Ra = 186.0$ nm), leading to relatively low conductivity (55.80 S/cm). Therefore, we can propose a possible mechanism in which hot-pressing drives the immigration of Ag flakes from inside to the outside surface, at the same time, interaction between Ag flakes and polymer binder determines the order arrangement of the Ag flakes on the surface." and "The morphology of nanocomposite conductor was observed by atomic force microscopes (AFM, BrukerIcon)." in Page 24.

For your convenience, the figures are also attached below.

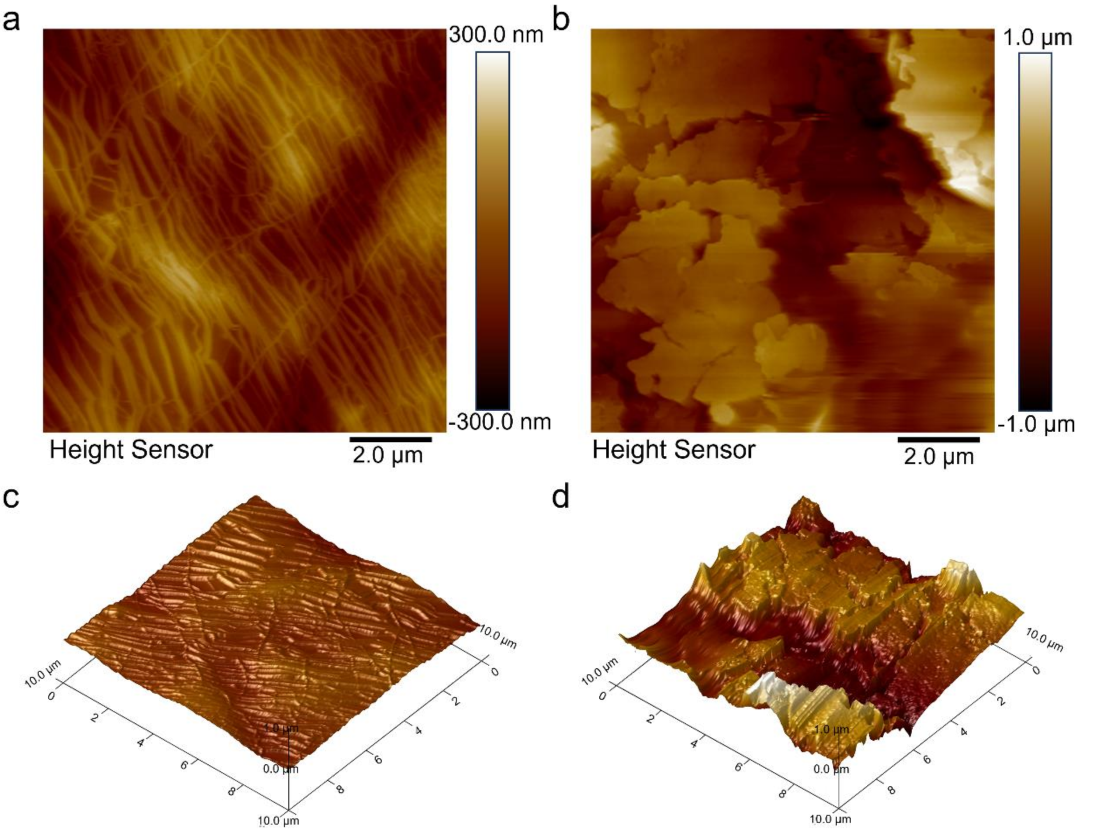

Figure S18 AFM (Atomic Force Microscope) images of (a) nanocomposite conductor in this work and (b) pPEGMA-Ag-70. AFM (Atomic Force Microscope) images of (c) nanocomposite conductor in this work (Ra = 40.6 nm) and (d) pPEGMA-Ag-70 (Ra = 186.0 nm). The test area is 1 μm × 1 μm.

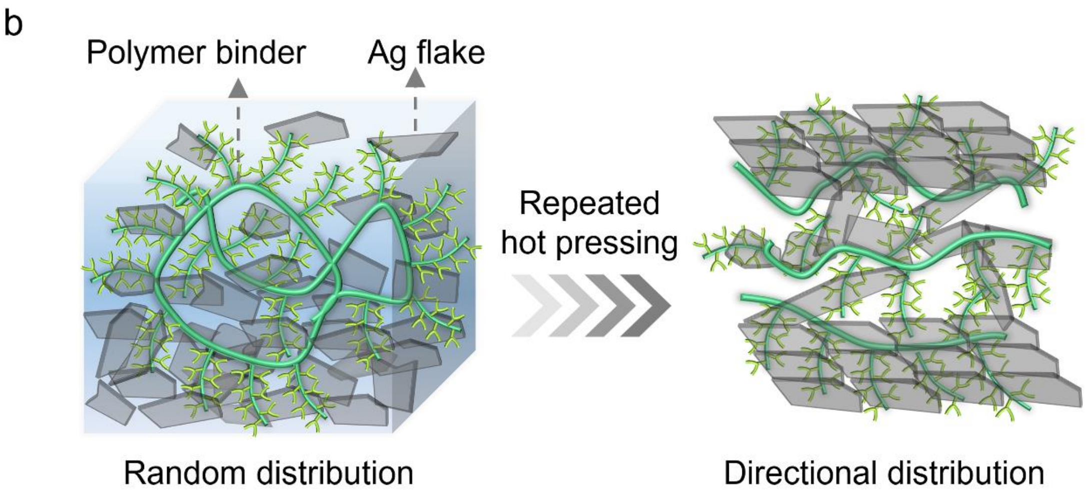

Figure 1 Fabrication of strain resilient nanocomposite conductor. (b) The fabrication process of strain-resilient conductors.

[2] There is no direct evidence confirming the presence of "coordination interaction" in which the lone pairs of the polymer binder stabilize the Ag flakes. If the authors intend to claim the presence of coordination interactions, they should additionally provide supporting data (e.g., FT-IR, XPS, Raman spectroscopy) along with the DSC curves in Fig. 2e.

Response: Thank you for your constructive comments. To verify the formation of coordination interactions, we performed FT-IR and X-ray photoelectron spectroscopy. As shown in Figure S7a, compared with the polymer pPEAOI, the absorption peaks of N-H, C=O, and C-O in the nanocomposite conductor pPEAOI-Ag-70 exhibit distinct red shifts from 3341 to 3306 cm^{-1}, 1638 to 1633 cm^{-1}, and 1095 to 1049 cm^{-1}, respectively. These shifts can be attributed to the donation of lone-pair electrons from nitrogen and oxygen atoms into the empty orbitals of Ag, which reduces the force constants of the corresponding bonds and further confirms the presence of coordination interactions between Ag and the polymer chains. Additionally, XPS (X-ray photoelectron spectroscopy) test was conducted to characterize the interaction between Ag flakes and polymer binder. As shown in Figure S7b, after the incorporation of pPEAOI, Ag $3d_{3/2}$ and Ag $3d_{5/2}$ peaks shifted from 374.55 and 368.55 eV to 374.27 and 368.27 eV, respectively. Correspondingly, the peaks of N1s and O1s shifted to higher binding energy due to the decreased electron density around N atoms and O atoms indicating the coordination interaction between Ag flakes and pPEAOI (Figure S7c, d). The relative discussion can be found on Page 7 in the revised manuscript as follows: "Additionally, Fourier Transform infrared (FT-IR) spectroscopy and X-ray photoelectron spectroscopy (XPS) test were conducted to confirm the coordination interaction between Ag flakes and polymer binder pPEAOI. As shown in Figure S7a, compared with the polymer pPEAOI, the absorption peaks of NH, C=O, and C-O in the nanocomposite conductor pPEAOI-Ag-70 exhibit distinct red shifts which could be attributed to the donation of lone-pair electrons from nitrogen and oxygen atoms into the empty orbitals of Ag. As shown in Figure S7b, after the incorporation of pPEAOI, the Ag $3d_{3/2}$ and Ag $3d_{5/2}$ peaks shifted from 374.55 and 368.55 eV to 374.27 and 368.27

eV, respectively. Correspondingly, the peaks of N 1s and O 1s shifted to higher binding energy indicating the coordination interaction between Ag flakes and pPEAOI (Figure S7c,7d).” and “X-ray photoelectron spectroscopy was conducted by XPS (Thermo Scientific K-Alpha, USA).”

For your convenience, the figures are also attached below.

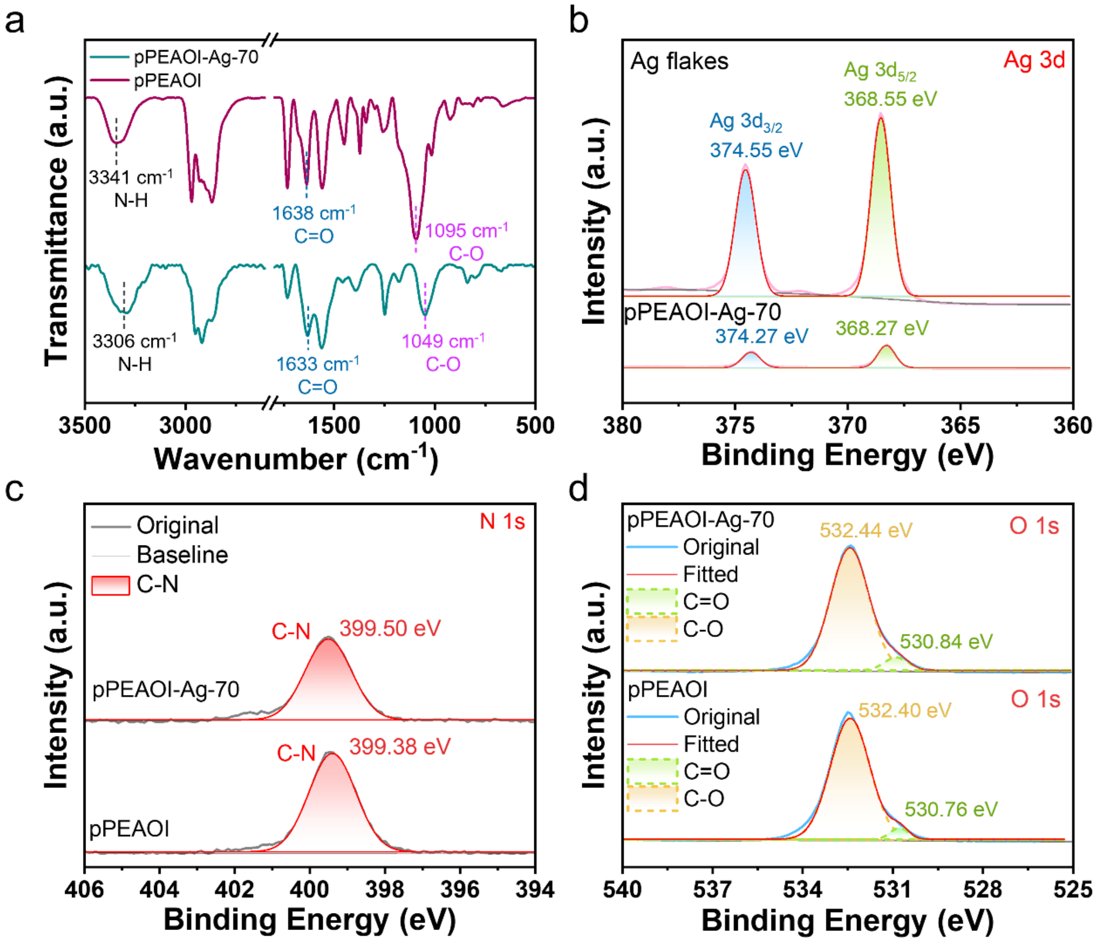

Figure S7 Conformation of coordination interaction between polymer binder and Ag flakes. (a) FT-IR spectroscopy of pPEAOI and pPEAOI-Ag-70. (b) Ag 3d core level XPS spectra of Ag flakes and pPEAOI-Ag-70. (c) N 1s and (d) O 1s core level XPS spectra of pPEAOI-Ag-70 and pPEAOI.

[3] The claim that a directional distribution is achievable seems to imply that the percolation threshold can be reduced. I am curious about the minimum filler concentration at which the percolation threshold is reached using this approach, and

whether it can be significantly lowered compared to other systems.

Response: Thank you for your insightful comments. As literature reported, the classical power law relates the conductivity of an unstretched material σ to V_f as follows:

$$\sigma = \sigma_0(V_f - V_c)^s$$

where σ_0 is the conductivity of the filler, V_f, and V_c are the volumetric fractions of the filler and percolation threshold at initial state, respectively, and *s* is the critical exponent. (*Compos. Sci. Technol.* **67**, 2114-2120 (2007).) To facilitate fitting, the equation was subjected to the following logarithmic transformation.

$$log\sigma = \log \sigma_0 + slog(V_f - V_c)$$

The lowest possible percolation threshold of nanocomposite conductor in this work was determined to be 11.4% volume fraction when the experimental data exhibited a perfect linear fit based on the equation ($R^2 = 0.9917$) (Figure S19). Compared to other Ag flakes nanocomposite conductors' system, the percolation threshold of nanocomposite conductors in this work is comparable to the best values with similar size of similar size of Ag flakes (Lowest percolation threshold for Ag flakes with size of 18.5 μm is 11.4% volume fraction. *Nature Materials* **16**, 834-840 (2017), the size of Ag flakes in this work is about 1~5 μm; Another nanocomposite conductor system published on ACS Nano has a percolation threshold at 12.12% volume fraction (*ACS Nano* **13,** 6531-6539 (2019)).

The relative discussion can be found on Page 14 in the revised manuscript as follows: "Consequently, the percolation threshold is reduced. According to the classical percolation threshold theory, the predicted percolation threshold for our nanocomposite system is as low as 11.4% volume fraction (Figure S19), which is comparable to analogous studies with Ag flakes of similar size [8,35]."

For your convenience, the figure is also attached below.

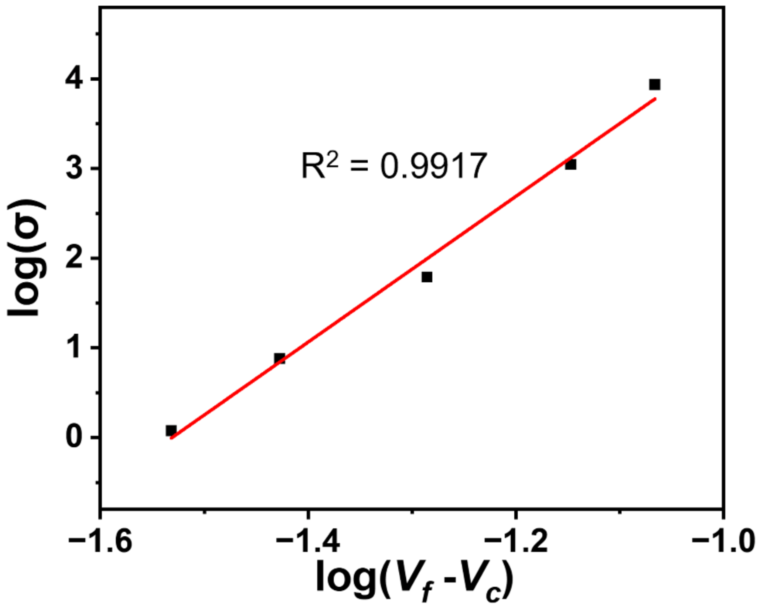

Figure S19 Experimental dependence of σ on V_f and its linearization according to the classical power-law relation of 3D percolation theory. Log plots of σ in respect to $\log(V_f - V_c)$ with a linear fit. The classical power law relates the conductivity of an unstretched material σ to V_f as follows:

$$\sigma = \sigma_0(V_f - V_c)^s$$

where σ_0 is the conductivity of the filler, V_f, and V_c are the volumetric fractions of the filler and percolation threshold at initial state, respectively, and s is the critical exponent[33]. To facilitate fitting, the equation was subjected to the following logarithmic transformation.

$$log\sigma = \log\sigma_0 + slog(V_f - V_c)$$

The lowest possible percolation threshold of nanocomposite conductor in this work was determined to be 11.4% volume fraction when the experimental data exhibited a perfect linear fit based on the equation (R^2 = 0.9917).

Reference:

33. Li, J., Kim, J.-K. Percolation threshold of conducting polymer composites containing 3D randomly distributed graphite nanoplatelets. *Composites Science and Technology* **67**, 2114-2120 (2007).

[4] In addition, it would be helpful to present the conductivity or sheet resistance depend on weight percent of Ag flake.

Response: Thank you for your valuable suggestions. As shown in Figure S8, we have presented the sheet resistance depends on weight percentage of Ag flakes.

The relative discussion can be found on Page 8 in the revised manuscript as follows: "The dependence of sheet resistance of nanocomposite conductors on the different contents of doped Ag flakes was further measured by Four-probe Tester HPS2526 (Figure S8)."

For your convenience, the figure is also attached below.

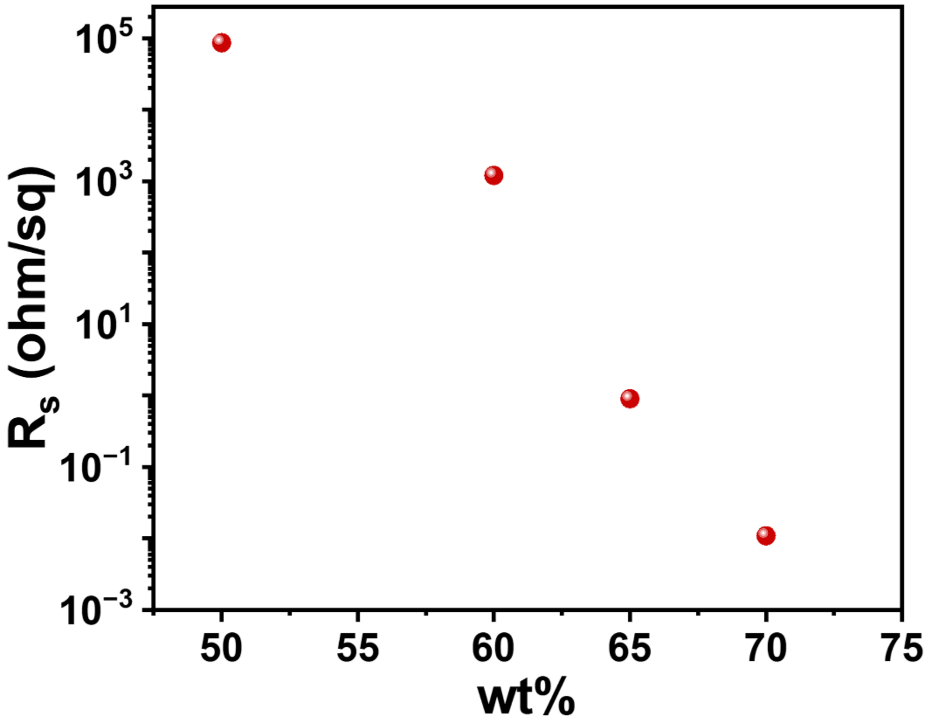

Figure S8 Sheet resistance depends on weight percentage of Ag flakes.

[5] In the mechanism section, additional comparisons with appropriate controls are needed—not as plasticizers for self-healing, but as systems that can help elucidate the underlying mechanism. This paper presents a siloxane-based control; however, siloxane is well known for its low chain friction and low Tg. Therefore, the observed low stress level and excellent self-healing performance are rather expected results. If the authors

intend to emphasize the effect of the brush polymer binder possessing abundant lone pairs, it is essential to include a comparison with another system containing ether chains but fewer lone pairs (e.g., PEGDMA).

Response: Thank you for your helpful suggestions. We fully agree with you that Siloxane exhibits lower stress and excellent self-healing performance. Thus, we have involved siloxane to lower the self-healing temperature. Moreover, pDMSOI had urea bonds but lacked ether chains, so it can prove the necessity of abundant lone pairs. According to your insightful suggestions, we use PEGMA as a control to further prove the contribution of urea bonds. Because the polymer of PEGDMA is so stiff and fragile, we have used PEGMA as an adhesive binder to conduct the experiment. The chemical structure is shown in Figure S17a. pPEGMA-Ag-70 was obtained after free radical polymerization by adding Ag flakes with a mass fraction of 70% to total mass. As shown in Figure S17b, the elongation of pPEGMA-Ag-70 is approximately 70%. During the stretching process, resistance of pPEGMA-Ag-70 increased from 39 Ω to 1246 Ω (Figure S17c). Moreover, the relative resistance change remains stable only within a strain range of 13%, and when the strain reaches 50%, the resistance reaches 179.86 Ω, correspondingly $R/R_0 = 4.61$ (Figure S17d, e). Apparently, pPEGMA-Ag-70 exhibited obvious degradation of mechanical property accompanied by highly unstable electrical property during mechanical information. To figure out the reason behind low conductivity of pPEGMA-Ag-70 (55.80 S/cm) compared to the pPEAOI-Ag-70 nanocomposite conductor with the same Ag contents, we conducted AFM (Atomic Force Microscope) experiment. As shown in Figure S18, the roughness (Ra) of pPEGMA-Ag-70 is 186.0 nm which is higher than that of nanocomposite conductor in this work (40.6 nm). Furthermore, pPEGMA can not drive the directional distribution of Ag flakes on the surface compared to heteroatoms-rich polymer binder due to the insufficient interaction between binder and Ag flakes. Overall, pPEGMA-Ag-70 exhibits poorer mechanical performance and unstable conductivity under deformation.

The relative discussion can be found on Page 14 in the revised manuscript as follows: "Additionally, poly(ethylene glycol) methyl ether methacrylate (PEGMA) with a

similar chemical structure which only has ether chains without urea bonds, was incorporated into the composite conductor as a polymer binder to demonstrate the necessity of urea bonds. The chemical structure of PEGMA is shown in Figure S17a. pPEGMA-Ag-70 was obtained after free radical polymerization by adding Ag flakes with a mass fraction of 70% to total mass. As shown in Figure S17b, the elongation of pPEGMA-Ag-70 is approximately 70%. During the stretching process, resistance of pPEGMA-Ag-70 increased from 39 Ω to 1246 Ω (Figure S17c). Moreover, the relative resistance change remains stable only within a strain range of 13%. When the strain reaches 50%, the resistance reaches 179.86 Ω, correspondingly R/R_0 = 4.61 (Figure S17d, e). Apparently, pPEGMA-Ag-70 exhibited obvious degradation of mechanical property accompanied by highly unstable electrical property during mechanical information due to the insufficient interaction between polymer binder and Ag flakes. Atomic Force Microscope (AFM) was utilized to further investigate the role of polymer binders in the migration and oriented distribution of Ag flakes. As shown in Figure S18, Ag flakes on the surface of pPEAOI-Ag-70 show obviously orderly packed distribution and lower roughness (Ra = 40.6 nm), which have significant positive effects to the continuous and stable conductive pathways, thus resulting in ultralow sheet resistance and high conductivity. On the contrast, Ag flakes on the surface of pPEGMA-Ag-70 did not exhibit similar order distribution and pPEGMA-Ag-70 showed higher roughness (Ra = 186.0 nm), leading to relatively low conductivity (55.80 S/cm)." and Materials synthesis and methods section as follows: "Poly(ethylene glycol) methyl ether methacrylate (PEGMA, n = 9) was purchased from TCI (Tokyo Chemical Industry Co., Ltd., Shanghai)" in Page 22; "The synthesis of pPEGMA-Ag-70 with PEGMA (n=9) was consistent with the above synthesis steps." in Page 23; "The morphology of nanocomposite conductor was observed by atomic force microscopes (AFM, BrukerIcon)." in Page 24; Moreover, for clearer comparison, the vertical axis in Figure 3b has been changed from time to strain.

For your convenience, figures are also attached below.

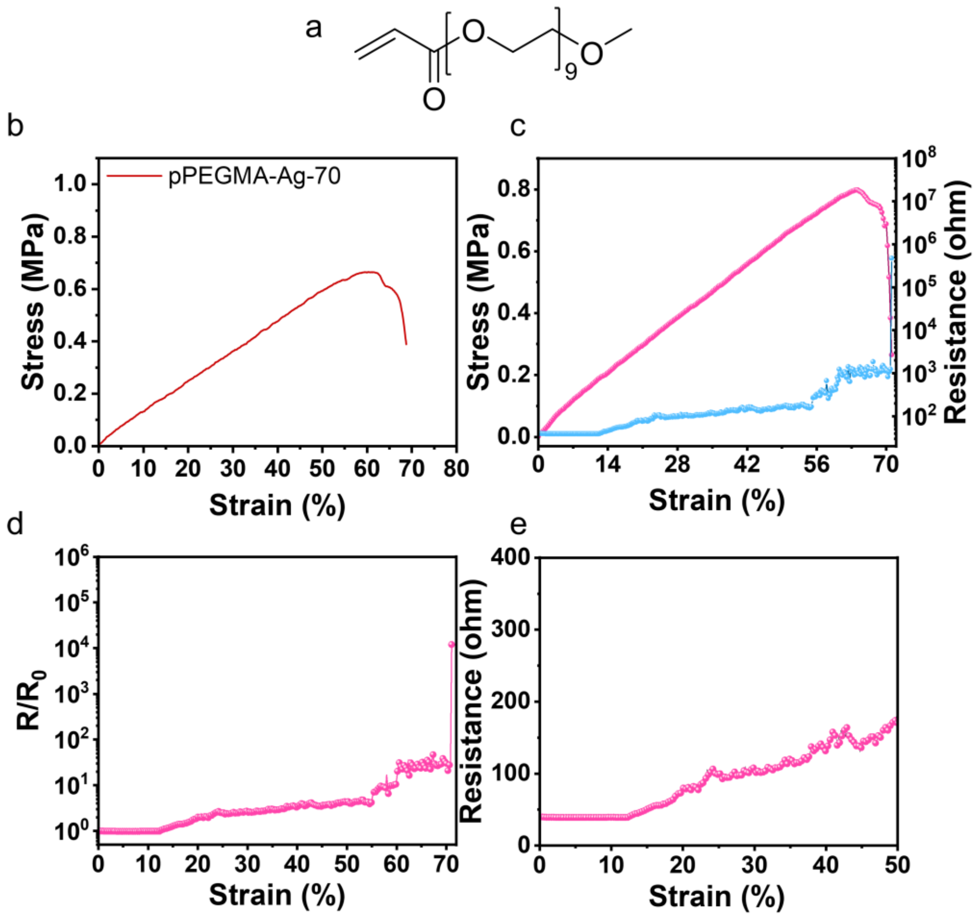

Figure S17 pPEGMA which only has ether chains without urea bonds as a control polymer binder to prove the positive effect of heteroatoms-rich polymer binder. (a) Chemical structure of PEGMA. (b) Stress-strain curves of pPEGMA-Ag-70 under speed of deformation at 10 mm/min. (c) Resistance change and (d) relatively resistance value (*R/R0*) change varies with strain during stretching. (e) Resistance change varies with strain within 50% range.

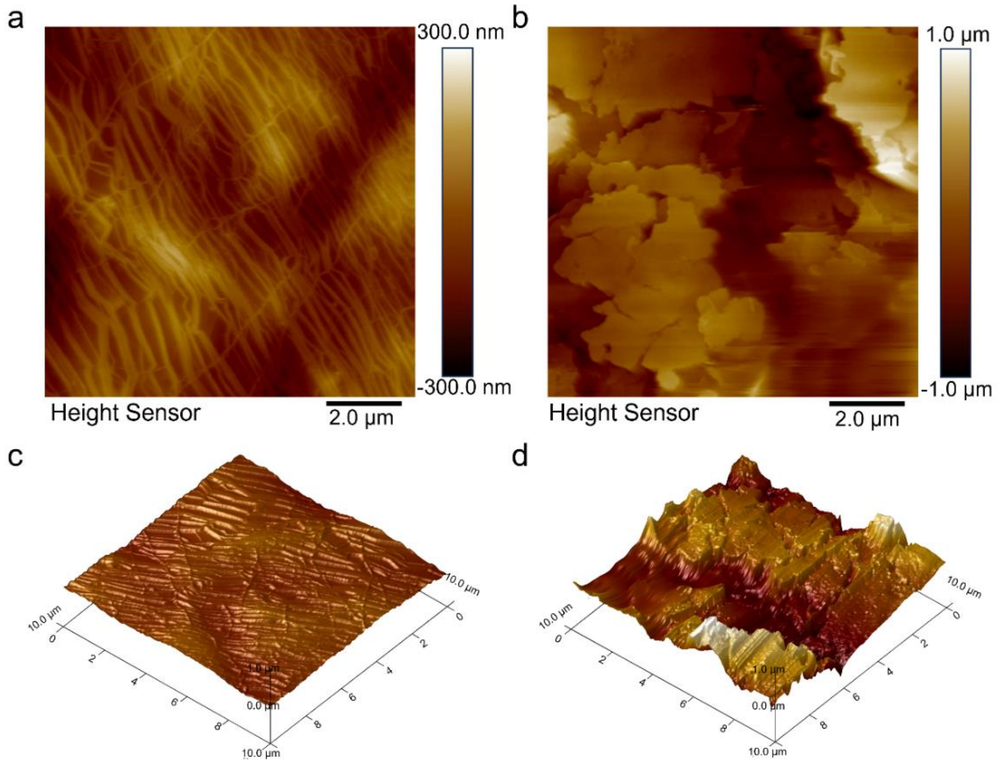

Figure S18 AFM (Atomic Force Microscope) images of (c) nanocomposite conductor in this work (Ra = 40.6 nm) and (d) pPEGMA-Ag-70 (Ra = 186.0 nm). The test area is 1 μm × 1 μm.

Reviewer #2 (Remarks to the Author):

Hou et al. presents a stretchable and self-healing conductor for stretchable electronics by introducing conductive fillers into a bottlebrush polymer binder. In this creeper-plant-inspired architecture, the conductive fillers (leaves) provide low resistance, whereas the polymer binders (branches) impart stretchability and self-healing capability. This design enabled the flexible conductor to maintain high conductivities (11574 S/cm) under repeated strain cycles (50 and 100%). Furthermore, hydrogen bonding within the bottlebrush chains allowed the conductors to self-heal after damage without a major loss in conductivity. The optimization of the heteroatom content in the bottlebrush to enhance the strain and fatigue resistance of the material while retaining self-healing properties was a nice experiment.

Response: Thank you for your positive comments. We have revised our manuscript thoroughly according to your suggestions, and all changes are highlighted in yellow color in the revised manuscript.

The authors note that there is a trade-off between “self-healing capability, conductivity, and flexible deformability of nanocomposite conductors”. Can the authors explain why there is trade-off between self-healing capability and flexibly deformability? My impression is that if something is easy to deform, it will be easier to “self-heal” as well.
Response: Thank you for your insightful suggestions. We are sorry for the misleading sentence. Indeed, if something is easy to deform, it will be easier to self-heal as well. Our intention is to highlight the trade-off between conductivity and flexible deformability, self-healing ability. The sentence is revised in Page 1 as follows: “However, nanocomposite conductors inherently suffer from a trade-off between conductivity and flexible deformability or self-healing ability.”; “In this way, the inherent trade-off issue between conductivity and flexible deformability or self-healing capability, would possibly be addressed.” in Page 4.

As well documented by the authors, there are many examples of self-healing conductors. My major critique of these work is that while some mechanical properties are improved, there are important mechanical properties that are lost. For instance, often the elongation at break are high and this work similarly shows this is true as well. However, for many materials that heal well, the elasticity is poor. It is very important to show the cycling data at different stain percentages to show there is hysteresis (or lack there of) at different strain rates. Otherwise, you can have a very stretchable material but cannot return back to its original shape. Indeed the authors show cycling data of the samples (such as figure 3d/e, it would be helpful to show real images rather than a cartoon here), but this is showing how the resistance changes rather than showing the mechanical properties. There is partial data (S16), but there is only a handful of cycles an an undefined strain rate. It is important to show the slower strain rates as this is where viscous properties are elucidated.

Response: Thank you for your insightful suggestions. We have replaced the cartoon with real optical images in Figure 3d and Figure 3e. Figure R1 showed the optical images. For your convenience, the revised Figure 3d and Figure 3e are attached below.

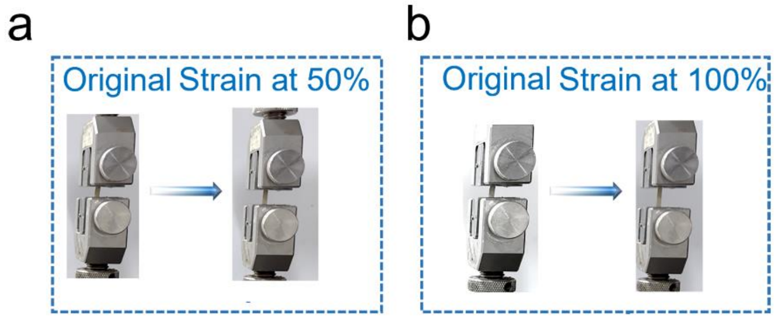

Figure R1 (a) Original and stretched (strain at 50%) optical images of pPEAOI-Ag-70. (b) Original and stretched (strain at 100%) optical images of pPEAOI-Ag-70.

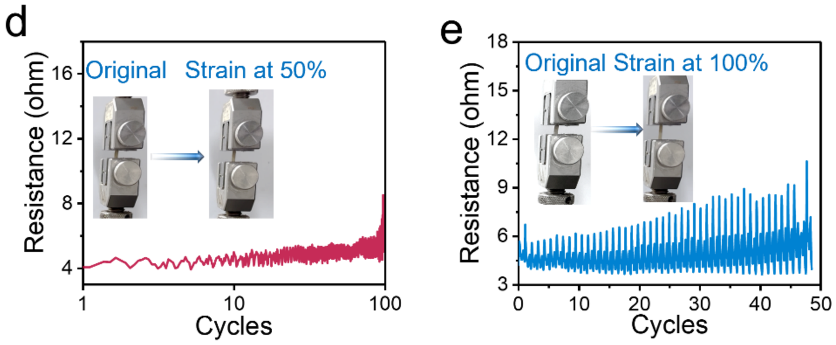

Figure 3 Change in resistance of pPEAOI- Ag-70 over (d) 100 stretch-relaxation cycles to 50% strain and (e) 48 stretch-relaxation cycles to 100% strain.

Furthermore, we have conducted cyclic tensile testing at varying rates to assess the elasticity of the nanocomposite conductor pPEAOI-DMSOI-50-Ag-70. According to literature reports (Nature **629**, 1047-1054 (2024)), wearable electronic devices can be subjected to stretching strains of up to 60% during use. Therefore, we investigated the viscoelastic behavior of pPEAOI-DMSOI-50-Ag-70 within 60% strain range at small deformation rates (2 mm/min and 3 mm/min). As shown in Figure S24a, when the strain rate was set at 2 mm/min, nanocomposite conductor pPEAOI-DMSOI-50-Ag-70 exhibited varying degrees of residual strain (for maximum strain at 20, 40, 60%, the residual strain is 8.4, 20, 24%, respectively.). Correspondingly, when the strain rate

was set at 3 mm/min, the residual strain of pPEAOI-DMSOI-50-Ag-70 at 20, 30, 40% strain is 7.4, 13.5 and 13.6%, respectively (Figure S24b). Overall, due to the addition of viscous component pDMSOI, nanocomposite conductor pPEAOI-DMSOI-50-Ag-70 exhibited hysteresis behavior at small strain rates.

Relative discussions can be found in Page 15 of the revised manuscript as follows: "As shown in Figure S23 and Figure S24, due to the addition of viscous component pDMSOI, nanocomposite conductor pPEAOI-DMSOI-50-Ag-70 exhibited hysteresis behavior at small strain rates."

For your convenience, figures are also attached below.

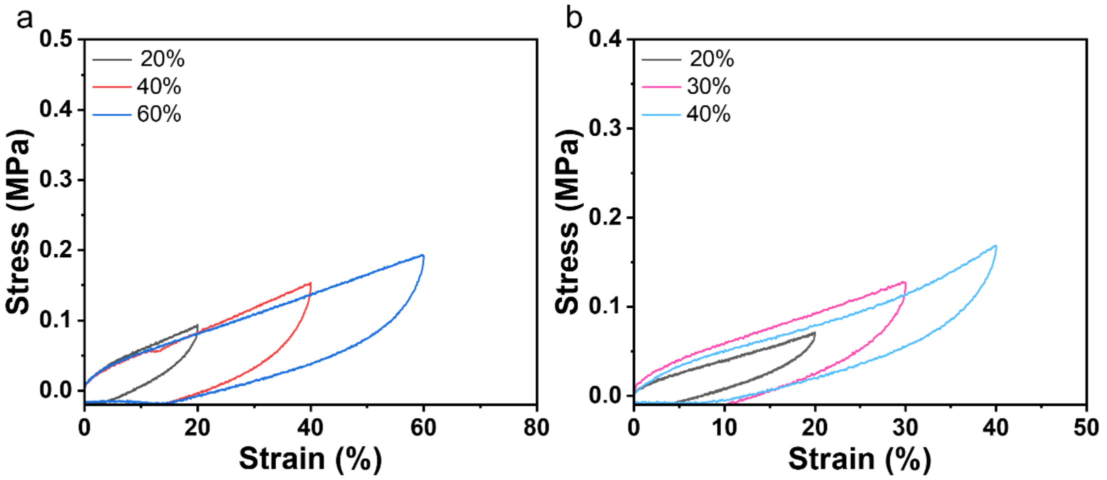

Figure S24 Curves of pPEAOI-DMSOI-50-Ag-70 stretched and relaxed under different strains at deformation speed of (a) 2 mm/min and (b) 3 mm/min.

Since the authors show some stretching videos, it would nice to show how the material relaxes.

Response: Thank you for your insightful suggestions. We have captured a video to demonstrate the relaxation of the material after stretching (Figure R2), which has been included into Movie S1 the stretchability and relaxation behavior of pPEAOI-DMSOI-50-Ag-70. Several representative frames were extracted from the video to present the results more intuitive.

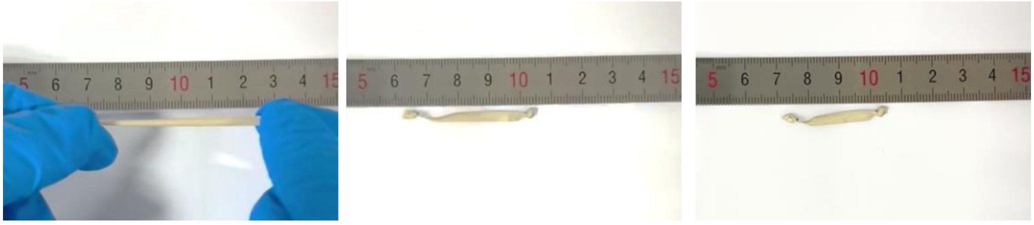

Figure R2 Optical images of pPEAOI-DMSOI-50-Ag-70 during stretched to relaxed.

Additionally, creep data is highly recommended as this paper should include full mechanical characterization data—this is easily collected on the same instruments. For me, this data is necessary for publication in Nature Communications.

Response: Thank you for your insightful suggestions. We totally agree with your point that creep is necessary due to concern about the long-term stability of the wearable electronics. We have performed additional creep experiments under both uniaxial stress (using a tensile testing machine) and shear stress (via a rheometer) to evaluate the material's long-term performance and stability. As shown in Figure S29a, under uniaxial constant stresses of approximately 0.10 MPa, 0.15 MPa, 0.20 MPa, and 0.30 MPa for 30 minutes at room temperature, the creep strain of the nanocomposite conductor pPEAOI-DMSOI-50-Ag-70 reached 13.20%, 19.91%, 42.27%, and 58.29%, respectively. Furthermore, rheological tests revealed that the material exhibits rapid self-repair capability when shear stress is removed. As also illustrated in Figure S29b, after the shear stress was released and the sample was allowed to recover for 30 minutes, the strain decreased from 7.03% to 0.52%, from 17.43% to 2.12%, and from 25.35% to 3.21%, respectively. Overall, pPEAOI-DMSOI-50-Ag-70 shows great potential in long-term wearable electronics due to the low creep strain value under small uniaxial stress (0.10 MPa) and excellent recoverability after shear creep.

On this basis, the manuscript has been revised in Page 16 as follows: "Furthermore, both tensile creep and shear creep experiments were conducted to evaluate the long-term stability of pPEAOI-DMSOI-50-Ag-70 as a component of wearable electronics under sustained tensile and shear stresses during service. As shown in Figure S29, it exhibited low creep strain value (13.20%) under small uniaxial stress (0.10 MPa) for

30 minutes and excellent recoverability after shear creep (from 25.35% to 3.21% at 3000 Pa), suggesting great potential in long-term stable wearable electronics."

For your convenience, the figure is also attached below.

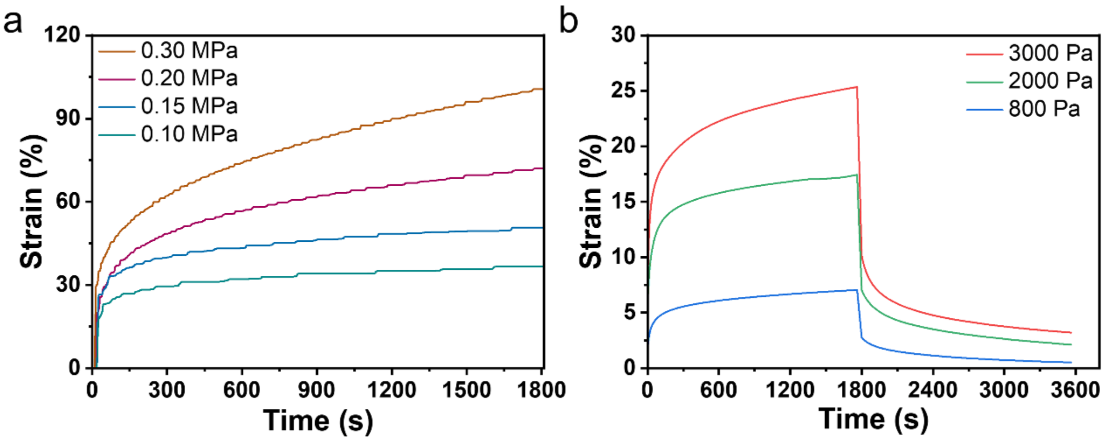

Figure S29 (a) Creep strain varies with time under different tensile stress. Under uniaxial constant stresses of approximately 0.10 MPa, 0.15 MPa, 0.20 MPa, and 0.30 MPa for 30 minutes at room temperature, the creep strain of the nanocomposite conductor pPEAOI-DMSOI-50-Ag-70 reached 13.20%, 19.91%, 42.27%, and 58.29%, respectively. (b) Curves of creep and recovery under different shear stress at 25 °C. After the shear stress was released and the sample was allowed to recover for 30 minutes, the strain decreased from 7.03% to 0.52%, from 17.43% to 2.12%, and from 25.35% to 3.21%, respectively.

For proper publications, please include the ppm shifts, integration, and j-couplings for small molecules in the NMR (SI).

Response: Thank you for your insightful suggestions. We have included the ppm shifts, integration and j-couplings for small molecules in the NMR. For your convenience, figures are attached below.

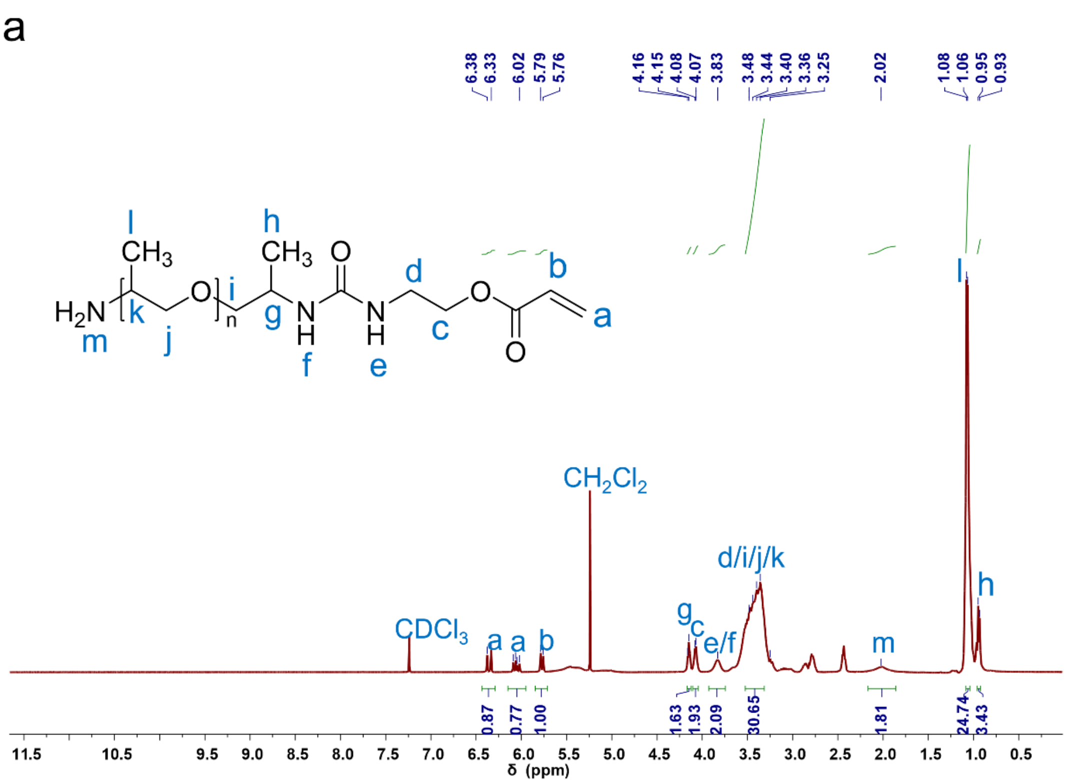

Figure S2a ^{1}H NMR spectrum of PEAOI: ^{1}H NMR (400 MHz, $CDCl_3$): δ (ppm) 6.36 (d, *J* = 17.3 Hz, 1H), 6.02 (m, 1H), 5.77 (d, *J* = 10.4 Hz, 1H), 4.16 (m, 1H), 4.07 (m, 2H), 3.83 (s, 2H), 3.40-3.25 (m, 22H), 2.02 (s, 2H), 1.07 (d, *J* = 5.7 Hz, 18H), 0.94 (d, *J* = 5.7 Hz, 3H).

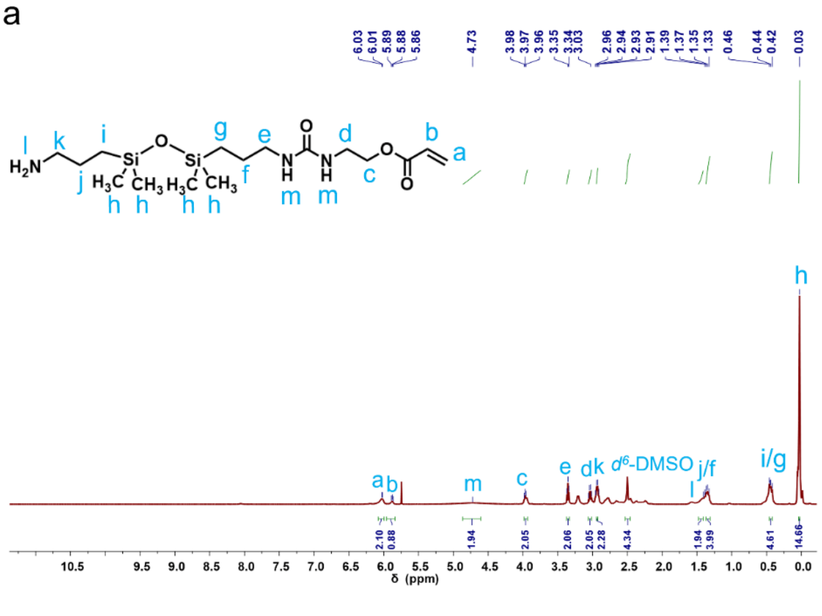

Figure S14a ^{1}H NMR spectrum of DMSOI: ^{1}H NMR (400 MHz, d^6-DMSO): δ (ppm) 6.02 (d, *J* = 5.2 Hz, 2H), 5.88 (t, *J* = 5.3 Hz 1H), 4.73 (s, 2H), 3.97 (m, 2H), 3.35 (t, *J*

= 5.8 Hz, 2H), 3.03 (m, 2H), 2.93 (m, 2H), 1.41 (s, 2H), 1.35 (m, 4H), 0.44 (m, 4H), 0.03 (s, 12H).

Lastly, I am unsure about the point about recyclable Ag flakes, as it is brought up at the end and it is not well motivated in the story of the manuscript. If it is included, then it would be nice to show that the flakes can be reintroduced into a new composite with equivalent electrical properties under mechanical deformation.

Response: Thank you for your kind suggestions. We have reintroduced the recycled Ag flakes into a new nanocomposite conductor. On this basis, the conductivity of the nanocomposite conductor made from recycled Ag flakes under mechanical force was recorded. As shown in Figure S39, the nanocomposite conductor made from the recycled Ag flakes maintained stable conductivity under punctured, twisted, bent and stretched damage or deformation.

Relative discussion could be found in the revised manuscript Page 20 as follows: "As shown in Figure S39, the nanocomposite conductor made from the recycled Ag flakes maintained stable conductivity under punctured, twisted, bent and stretched damage or deformation."

For your convenience, the figure is also attached below.

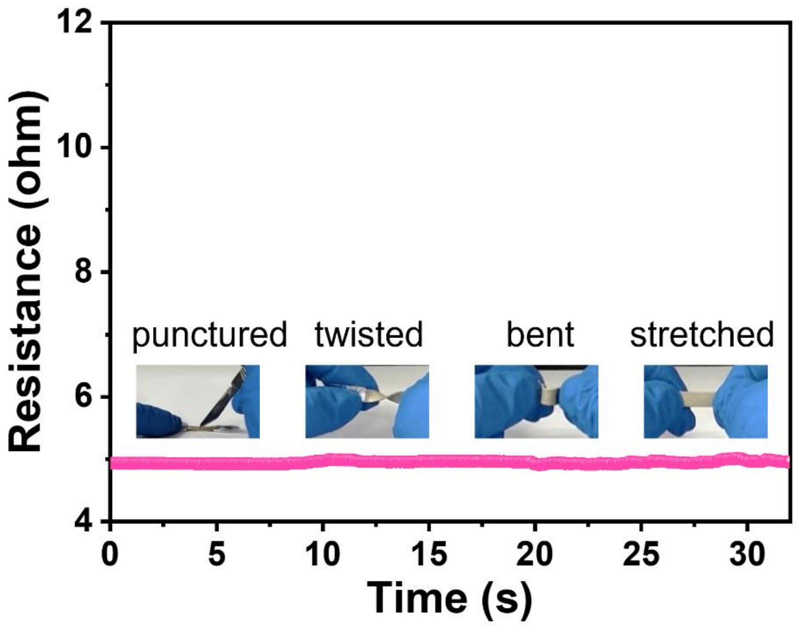

Figure S39 Real-time resistance of nanocomposite conductor made from recycled Ag flakes under mechanical damage or deformation.

Reviewer #3 (Remarks to the Author):

This manuscript reports the design of a creeper-inspired, self-healable and highly stretchable conductive nanocomposite based on Ag flakes embedded in a viscoelastic brush polymer binder. By hot pressing, the authors induce a surface-enriched, multilayer distribution of Ag flakes, achieving an ultralow initial sheet resistance (~10.8 mΩ sq^{-1}) and high conductivity. The composite exhibits large mechanical stretchability (up to ~700%), partial mechanical self-healing, and recovery of electrical continuity after damage. Leveraging these properties, the authors fabricate a stretchable, self-healable antenna operating at 2.4 GHz and demonstrate a wireless e-skin system capable of Bluetooth communication over tens of meters under small mechanical deformation.

Response: Thank you for your positive comments. We have revised our manuscript thoroughly according to your suggestions, and all changes are highlighted in yellow color in the revised manuscript.

Although the experimental effort is substantial, the work suffers from fundamental shortcomings in performance benchmarking, terminology accuracy, and application relevance. Moreover, the emphasis on bio-inspired design is overstated – there is only superficial similarity between the material microstructure and the Virginia Creeper plant and not enough scientific support for more fundamental commonalities between the two. While I think that this paper is a potentially valuable contribution to the field, the authors should address the following issues for this work to merit publication:

Response: Thank you for your insightful suggestions and sorry for the misleading caused by inappropriate adscription. According to your concerns, we have used comparing experiments to reveal the benefits of creeper-like structure and terminology accuracy is improved in the revised manuscript. We agree with the reviewer's point that there is only some similarity between the material microstructure and the Virginia Creeper. Therefore, we have deleted the text about bio-inspired concepts. And we just discussed the similarity between the material microstructure and the Virginia creeper. The manuscript is revised in Page 1 as follows: "Herein, a strain resilient and self-healing conductor (with ultralow sheet resistance of 10.8 mΩ/sq) was fabricated based on a well-designed polymer binder."

1. The creeper-inspired design with directionally accumulated distribution of Ag flakes is presented as a key concept. While SEM and EDS data confirm surface-enriched filler distribution after hot pressing, the manuscript does not provide a direct comparison between this structure and a randomly distributed Ag-flake composite with otherwise similar composition. To fully support the claimed advantage of this directional architecture, it would be valuable to demonstrate how it improves electrical stability, hysteresis, or durability relative to a control sample without induced directional migration. Without such comparison, the benefits of the creeper-inspired design remain unrevealed.

Response: Thank you for your helpful suggestions. To directly prove the advantage of the directional architecture, we have replaced hot-pressing by ordinary cold-pressing during the post-treatment to fabricate a nanocomposite conductor with Ag flakes

without induced directional migration named as pPEAOI-DMSOI-50-Ag-70-cold-pressing. As shown in Figure S28, pPEAOI-DMSOI-50-Ag-70-cold-pressing exhibited obvious electrical hysteresis after 1000 cycles deformation at strain of 50% ($\Delta R/R_0$ = 0.30, where $\Delta R = R\text{-}R_0$, R is the real-time resistance and R_0 is the initial resistance.) compared to pPEAOI-DMSOI-50-Ag-70 (of which $\Delta R/R_0$ = 0.06). Furthermore, with increasing cycle numbers, pPEAOI-DMSOI-50-Ag-70-cold-pressing exhibited a more pronounced increase in R/R_0 with strain (maximum R/R_0 = 1.92 compared to which pPEAOI-DMSOI-50-Ag-70 is stably around 1.15), and a significantly worse recovery of resistance when the strain is removed. Overall, due to the surface-enriched filler distribution after hot pressing, pPEAOI-DMSOI-50-Ag-70 have better electric stability and lower electrical hysteresis compared to pPEAOI-DMSOI-50-Ag-70-cold-pressing which directly confirm the benefits of the surface-enriched filler distribution achieved through hot pressing.

The relative discussion can be found on Page 15 in the revised manuscript as follows: "To directly prove the advantage of the directional architecture, we have replaced hot-pressing with ordinary cold pressing during the post-treatment. A nanocomposite conductor with Ag flakes but without induced directional migration was fabricated and named as pPEAOI-DMSOI-50-Ag-70-cold-pressing. As shown in Figure S28, pPEAOI-DMSOI-50-Ag-70-cold-pressing exhibited a more pronounced increase in R/R_0 with strain (maximum R/R_0 = 1.92, compared to 1.15 for pPEAOI-DMSOI-50-Ag-70), a significantly worse recovery of resistance when the strain is removed, and obvious electrical hysteresis after 1000 cycles deformation at strain of 50% ($\Delta R/R_0$ = 0.30)."

For your convenience, the figures are also attached below.

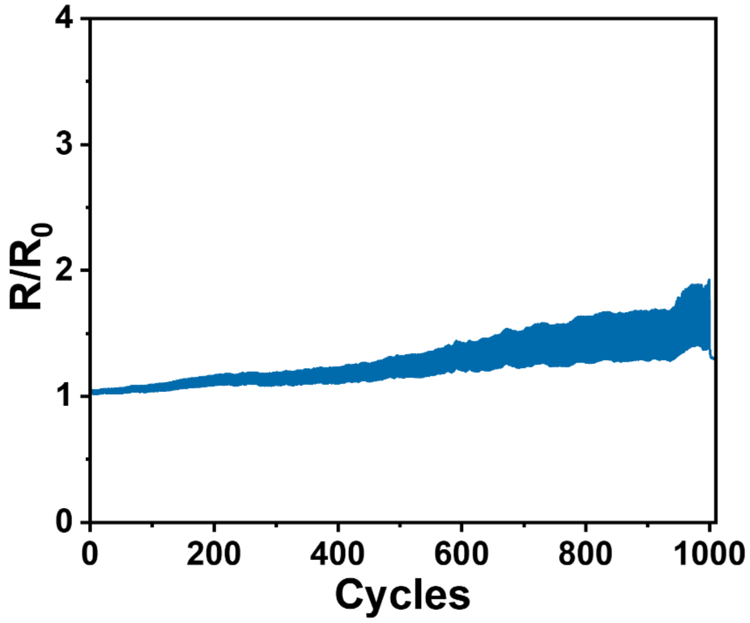

Figure S28 Change in relative resistance of pPEAOI-DMSOI-50-Ag-70-cold pressing over 1000 stretch-relaxation cycles to 50% strain.

2. The authors report that for "pPEAOI-DMSOI-50-Ag-70", the electrical resistance increases to approximately "64 Ω at 700 % strain" (as shown in Fig. 4e and Fig. S15). While the corresponding normalized resistance change ($R/R_0 \approx 15.8$) is lower than many previously reported stretchable conductors, the absolute resistance value is still relatively high. Such resistance levels seem non-trivial for practical applications including RF interconnects requiring low noise and minimal RC delay. Therefore, it would be helpful if the authors could clarify the operational strain condition for practical electronic and RF applications and distinguish it from the extreme-strain mechanical survivability demonstrated in tensile tests.

Response: Thank you for your insightful suggestions. As reported in a literature published on Nature (*Nature* **629**, 1047-1054 (2024)), natural physical motions of the human wrist can induce strains of up to 30% in skin-interfaced systems, and for EEG monitor, the flexible electronics stretches on wearing lead to a strain of up to 50%. In addition, the headband-like wearable electronics stretches up to 50% when worn on the head of an adult. Some other works related to stretchable radio frequency antenna usually set the strain to 100% to evaluate stability of data transmission (*Nature*

Communication **11**, 5629 (2020); *Advanced Materials* **36**, 2400103 (2024)). Moreover, other papers about stretchable electronics also focus on the performance under strain of 100% such as Highly stretchable polymer semiconductor films through the nanoconfinement effect (*Science* **355**,59-64 (2017)).

Therefore, according to your suggestion, we have revised the manuscript in Page 15 as follows: "As reported, natural human motions can induce strains up to 50% in skin-interfaced wearable systems during practical operation[49]. Therefore, to evaluate the stability of the nanocomposite conductor in practical applications, long-term cyclic stability testing was performed at a fixed tensile strain of 50%. As shown in Figure 4f, after 1000 cycles at 50% strain, R/R_0 stays below 1.15, and resistance remains under 4.25 Ω (Figure S25), with only minor surface microcracks observed (Figure S26). Furthermore, maintaining a low resistance is essential for stretchable RF antennas, requiring high stability under strains up to 100% [50-52]. As shown in Figure S27, even at 100% strain, R/R_0 stays below 1.13 over 70 cycles, with resistance under 4.28 Ω, showing significantly improved performance over pPEAOI-Ag-70."

References:

49. Kim, S.H. *et al.* Strain-invariant stretchable radio-frequency electronics. *Nature* **629**, 1047-1054 (2024).

50. Xu, J. *et al.* Highly stretchable polymer semiconductor films through the nanoconfinement effect. *Science* **355**, 59-64 (2017).

51. Zhang. Y. *et al.* High precision epidermal radio frequency antenna via nanofiber network for wireless stretchable multifunction electronics. *Nature Communications* **11**, 5629 (2020).

52. Sun. Y. *et al.* Projection stereolithography 3D printing high-conductive hydrogel for flexible passive wireless sensing. *Advanced Materials* **36**, 2400103 (2024).

3. The term "strain-insensitive conductivity" is used repeatedly throughout the manuscript. However, the experimental data indicate that:

• At "200 % strain, $R/R_0 \approx 1.22$," which is reasonably supports this description.

• At "700 % strain, $R/R_0 \approx 15.8$," representing a significant resistance increase. Although this behavior is still impressive relative to prior work, describing the electrical conductivity of the composite as "strain-insensitive across the entire strain range" appears a little bit misleading. The authors are encouraged to clearly define the strain range over which the material can be considered electrically "strain-insensitive".

Response: Thank you for your suggestive advice. We are sorry for the misleading description. Aligning with your comments, we have investigated the definition of strain-insensitive conductivity to enhance the precision of the description. On this basis, the manuscript has been revised as: "Overall, pPEAOI-Ag-70 not only has strain-insensitive conductivity under 130% stretch (maximum R/R_0 = 1.20) but also can maintain high conductivity after multiple cyclic stretching under large strain conditions." in Page 10; "Besides stable electrical performance, pPEAOI-Ag-70 also exhibits self-healing behavior of electrical property and mechanical property." in Page 11; "Overall, benefiting from the strong interaction between polymer binder pPEAOI and Ag flakes, the conductive nanocomposite pPEAOI-Ag-70 exhibited superior electrical performance and self-healing property." in Page 11; "The interconnected conductive pathways, restrained by the polymer binder, avoid sliding and disconnecting under stretching conditions, thereby ensuring highly strain-insensitive electrical performance under nearly 200% strain and maintaining its electrical integrity even when subjected to strains exceeding 700%." in Page 22; "The fabrication strategy of stretchable conductors with superior electrical performance and self-healing ability promises intrinsically self-healing wireless wearable electronics." in Page 22. Moreover, the strain insensitivity range shown in Figure 4i has been revised based on the definition of strain-insensitive electrical performance.

For your convenience, the figure is also attached below.

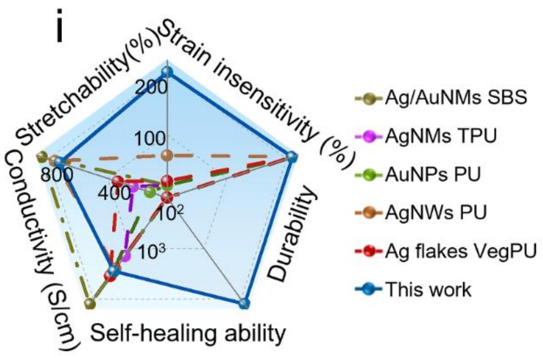

Figure 4 Mechanism and performance optimization of Ag flakes-based nanocomposite conductors. (i) Comparison of comprehensive property of pPEAOI-DMSOI-50-Ag-70 to analogous reported metallic nanocomposites[17,21,24,27,62].

4. Reporting on sample geometry and conductivity measurement under strain are unclear. While resistance–strain curves are shown, the manuscript does not clearly specify the sample geometry and dimensions used for resistance measurements. Because geometric elongation alone can significantly affect resistance, it would be important to clarify how these factors were accounted for. Providing electrical conductivity or resistivity data under strain, or explicitly stating measurement assumptions, would strengthen the quantitative interpretation.

Response: Thank you for your suggestive advice. The sample geometry used for resistance measurement is rectangular block. On this basis, the conductivity during stretching was measured according to the method literatures reported under the assumption that the total volume of the nanocomposite conductor does not change (*Nature* 500, 59-63 (2013); *Nature Materials* 16, 834-840 (2017)). Conductivity-strain characteristics of the nanocomposite conductor pPEAOI-Ag-70 and pPEAOI-DMSOI-50-Ag-70 are shown in Figure S10 and Figure S22, respectively.

The relative discussion can be found in the revised manuscript as follows: “Corresponding conductivity-strain characteristics of the nanocomposite conductor pPEAOI-Ag-70 are shown in Figure S10.” in Page 10 and “Corresponding conductivity-strain characteristics of pPEAOI-DMSOI-50-Ag-70 are shown in Figure S22.” in Page 15.

For your convenience, the figure is also attached below.

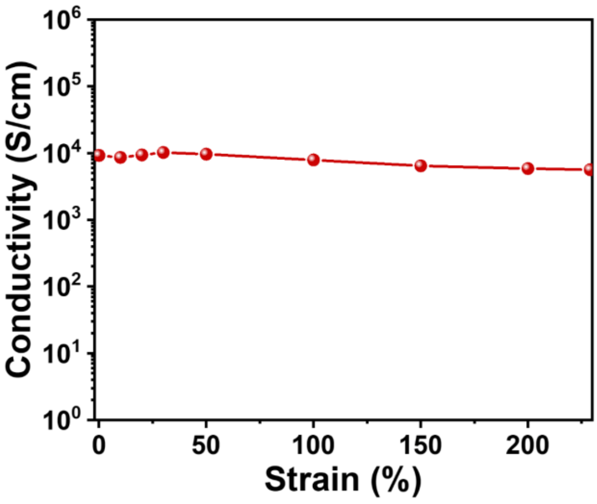

Figure S10 Conductivity-strain characteristics of nanocomposite conductor pPEAOI-Ag-70.

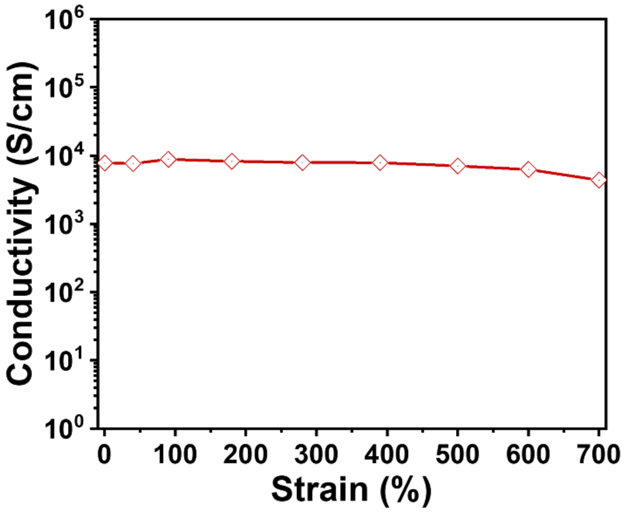

Figure S22 Conductivity-strain characteristics of nanocomposite conductor pPEAOI-DMSOI-50-Ag-70.

5. The presentation of the supplementary movies is not sufficiently quantitative or informative. While the videos qualitatively illustrate stretchability and electrical continuity, they lack clear annotations of the data. Without these elements, the supplementary movies do not meaningfully support the quantitative claims made in the manuscript and should be improved to provide scientifically rigorous, data-related

visualization.

Response: Thank you for your suggestive advice. We have added more annotations of data in supporting movies and retaken Movie S2.

Reviewer #1 (Remarks to the Author):

Authors have addressed my comments appropriately. I am satisfied with the revisions and recommend this work for publication in Nature Communications.

Response: Thank you very much for your time and effort in reviewing our manuscript. Your insightful comments and suggestions prompted us to conduct additional experiments and provide more detailed clarifications, which have significantly strengthened the manuscript. We are grateful for your valuable contribution to improving the quality of our study.

Reviewer #2 (Remarks to the Author):

My comments are adequately addressed; however, after reading the other reviewers comment, I must also say I think the analogy to the plant is a stretch. I recommend it is removed. I almost made the same comment but was on the fence.

Response: We appreciate your acknowledgment that our previous revisions adequately addressed your concerns. Regarding your specific suggestion about the plant analogy, we have carefully considered your perspective. We agree that this analogy may not be essential to the core scientific argument. Therefore, we have revised Figure 1 and significantly reduced the prominence of the plant analogy throughout the manuscript.

Reviewer #3 (Remarks to the Author):

The authors have addressed all my previous concerns through additional experiments and detailed clarifications. The revised manuscript is significantly strengthened and now I think this meets the standards of Nature Communications. I recommend its publication.

Response: Thank you very much for your time and effort in reviewing our manuscript. We sincerely appreciate your constructive feedback throughout the review process, which has significantly improved the quality and clarity of our study.
